# Clinical guidelines for primary sclerosing cholangitis 2017

**DOI:** 10.1007/s00535-018-1484-9

**Published:** 2018-06-27

**Authors:** Hiroyuki Isayama, Susumu Tazuma, Norihiro Kokudo, Atsushi Tanaka, Toshio Tsuyuguchi, Takahiro Nakazawa, Kenji Notohara, Suguru Mizuno, Nobuhisa Akamatsu, Masahiro Serikawa, Itaru Naitoh, Yoshiki Hirooka, Toshifumi Wakai, Takao Itoi, Tomoki Ebata, Shinji Okaniwa, Terumi Kamisawa, Hiroki Kawashima, Atsushi Kanno, Keiichi Kubota, Masami Tabata, Michiaki Unno, Hajime Takikawa, PSC guideline committee Members: Ministry of Health, Labour and Welfare (Japan) Research Project, The Intractable Hepatobiliary Disease Study Group

**Affiliations:** 1 Department of Gastroenterology, Graduate School of Medicine, Juntendo University, Tokyo, Japan; 2 Department of General Internal Medicine, Graduate School of Biomedical and Health Sciences, Hiroshima University Hospital, 1-2-3, Kasumi, Minami-ku, Hiroshima, 734-8551 Japan; 3 Department of Surgery, National Center for Global Health and Medicine, Tokyo, Japan; 4 Department of Medicine, Teikyo University School of Medicine, Tokyo, Japan; 5 Department of Medicine and Gastroenterology, Chiba University, Chiba, Japan; 6 Department of Gastroenterology, Japanese Red Cross Nagoya Daini Hospital, Nagoya, Japan; 7 Department of Anatomic Pathology, Kurashiki Central Hospital, Kurashiki, Japan; 8 Department of Gastroenterology, Graduate School of Medicine, The University of Tokyo, Tokyo, Japan; 9 Artificial Organ and Transplantation Division, Department of Surgery, Graduate School of Medicine, The University of Tokyo, Tokyo, Japan; 10 Department of Gastroenterology and Metabolism, Applied Life Sciences, Institute of Biomedical and Health Sciences, Hiroshima University, Hiroshima, Japan; 11 Department of Gastroenterology and Metabolism, Graduate School of Medical Sciences, Nagoya City University, Nagoya, Japan; 12 Department of Endoscopy, Nagoya University Hospital, Nagoya, Japan; 13 Division of Digestive and General Surgery, Niigata University Graduate School of Medical and Dental Sciences, Niigata, Japan; 14 Department of Gastroenterology and Hepatology, Tokyo Medical University, Tokyo, Japan; 15 Division of Surgical Oncology, Department of Surgery, Nagoya University Graduate School of Medicine, Nagoya, Japan; 16 Department of Gastroenterology, Iida Municipal Hospital, Nagano, Japan; 17 Department of Internal Medicine, Tokyo Komagome Metropolitan Hospital, Tokyo, Japan; 18 Department of Gastroenterology, Nagoya University Graduate School of Medicine, Nagoya, Japan; 19 Division of Gastroenterology, Tohoku University Graduate School of Medicine, Sendai, Miyagi Japan; 20 Second Department of Surgery, Dokkyo Medical University, Tochigi, Japan; 21 Department of Surgery, Matsusaka Central General Hospital, Matsusaka, Mie Japan; 22 Department of Surgery, Tohoku University Graduate School of Medicine, Sendai, Miyagi Japan

**Keywords:** Primary sclerosing cholangitis, Sclerosing cholangitis, Benign biliary stricture, Cholestasis, Guidelines

## Abstract

**Background:**

Primary sclerosing cholangitis (PSC) is relatively rare disease and pathogenesis and methods of treatments were still not established. Then, we had conducted the making clinical guidelines to manage patients with PSC based on the literature review and expert opinions. These clinical guidelines were made for the medical doctors on the management of PSC, except child case of PSC.

**Methods:**

We had employed modified Delphi method. The production committee decided guidelines, strength of recommendations and evidence level after reviewed literatures systematically, and The Expert panel evaluated those. The Scientific Committee of the Japan Biliary Association (JBA) evaluated revised guidelines, and the Public comments were collected on web site of JBA.

**Results:**

We had made 16 guidelines about epidemiology/pathophysiology, diagnostics, therapy and prognosis. Also, we had made both diagnostic and therapeutic flow chart.

**Conclusions:**

We hope that these guidelines will contribute to the improvement and development of the medical care of PSC.

## Introduction

Although most of the studies concerning primary sclerosing cholangitis (PSC) are published in Western countries, it is often difficult to adapt these findings to actual medical practice in Japan as many of the current conditions in Japan differ from those overseas. However, little evidence has been published in Japan. As a great deal of confusion is likely to arise at clinical settings because of this situation, we believe that the formal clinical guidelines are required for PSC.

As mentioned above, the level of evidence regarding this disease is not particularly high. Thus, in order to ensure that these guidelines reflect the consensus of experts in the field, we utilized Formal Consensus Development (the Delphi Method). As the Delphi Method combines published evidence with expert opinion in order to more objectively reflect the opinions of experts, we believe that it is the best method for the creation of clinical guidelines for PSC. The level of evidence and the level of recommendation were determined according to a grade system. The criteria used to determine the level of evidence and strength of the recommendations utilized in these guidelines is presented on the following page. These clinical guidelines were made for the medical doctors on the management of PSC, except child case of PSC. Japanese Ministry of Health, Labor and Welfare Research project supported this activity, and The Intractable Hepatobiliary Disease Study Group members contributed to make this guidelines.

## Methods

Three committees had made these clinical guidelines of PSC: Production committee, expert panel and Scientific Committee of the Japan Biliary Association as evaluation committee (Table 1). Production committee (7 members and 4 assistant members) includes 8 hepatobiliary gastroenterologists (including 5 endoscopists), 2 surgeons (Hepato-pancreato-biliary and liver transplant) and 1 pathologist.

**Table 1:** Committee composition

Production committee
Susumu Tazuma (Chair), Hiroyuki Isayama, Norihiro Kokudo, Atsushi Tanaka
Toshio Tsuyuguchi, Takahiro Nakazawa, Kenji Notohara
Production Assistant: Nobuhisa Akamatsu, Masahiro Serikawa, Itaru Naito
Suguru Mizuno
Panel of Delphi method experts
Atsushi Tanaka(Chair), Hiroyuki Isayama, Norihiro Kokudo, Susumu Tazuma
Toshio Tsuyuguchi, Takahiro Nakazawa, Kenji Notohara
Evaluation Committee (Scientific Committee of the Japan Biliary Association)
Yoshiki Hirooka (Chair), Toshifumi Wakai, Takao Itoi, Tomoki Ebata, Shinji Okaniwa,
Terumi Kamisawa, Hiroki Kawashima, Atsushi Kanno, Keiichi Kubota, Masami Tabata, Michiaki Unno

The first author (H.I) proposed the lists of clinical questions (CQ) about the management of PSC through the internet, and the Production Committee members discussed it by e-mail. Based on the discussion, the first and the corresponding author (H.I and S.T) decided the list of CQs (Table 2). The method of searching the available literature is also shown in the following sentences. In brief, we utilized basic keyword “PSC” and additional individual keywords for each CQ in our search of PubMed, Cochrane library and Ichushi-Web (Japanese journal searching engine). Searches were performed by each CQ manager and additional individual search keywords and primary numbers of hitting for these keywords are described in Table 3. The Production Committee members had reviewed the literatures systematically and had made the reference list. Each guideline, strength of recommendation and evidence level were decided by each CQ manager (Table 4). Those were once discussed using e-mail, and in face-to-face meeting of Production Committee.

**Table 2:** List of CQs and members in charge

Epidemiology/pathophysiology
CQ1. What is the pathophysiology of primary sclerosing cholangitis? (Tazuma)
CQ2. What is the epidemiology of primary sclerosing cholangitis? (Tanaka)
CQ3. What are the features of primary sclerosing cholangitis in Japan? (Tanaka)
CQ4. What are the risk factors of primary sclerosing cholangitis? (Tazuma)
Diagnostics
CQ5. What are the symptoms that suggest primary sclerosing cholangitis? (Isayama)
CQ6. What are the characteristic blood test findings for primary sclerosing cholangitis? (Nakazawa)
CQ7. What are the diagnostically useful imaging findings for primary sclerosing cholangitis? (Nakazawa)
CQ8. On what type of cases is endoscopic retrograde cholangiopancreatography (ERCP) performed? (Nakazawa)
CQ9. Is liver biopsy useful for the diagnosis of primary sclerosing cholangitis? (Notohara)
CQ10. How is this disease differentiated from cholangiocarcinoma and how are complications diagnosed? (Tsuyuguchi)
Therapy
CQ11. What pharmacotherapies are effective? (Isayama)
CQ12. How is itching treated? (Isayama)
CQ13. What are the indications for and methods of utilizing biliary drainage? (Tsuyuguchi)
CQ14. What is the optimal timing and indications for liver transplantation (Kokudo)
Prognosis
CQ15. What is the prognosis for primary sclerosing cholangitis? (Tanaka)
CQ16. What are the complications associated with primary sclerosing cholangitis? (Tsuyuguchi)

**Table 3:** Additional keywords of each CQ and number of hits in searching process

No.	Additional keywords	Number of hits		
		PubMed	Cochrane	Ichushi-Web
CQ1	Etiology, pathogenesis	202	6	48
CQ2	Epidemiology	269	6	14
CQ3	Japan	64	6	48
CQ4	Pathology, causes, pathophysiology	388	6	19
CQ5	Diagnosis, symptoms, cohort studies, epidemiology	400	6	20
CQ6	Cholestasis, biochemical test, biliary enzymes, liver enzymes, autoantibody, diagnosis	130	69	69
CQ7	Imaging diagnosis	412	111	207
CQ8	Endoscopic retrograde cholangiopancreatography, magnetic resonance cholangiopancreatography	153	35	11
CQ9	Liver biopsy	117	20	74
CQ10	Cholangiocarcinoma, diagnosis	557	6	84
CQ11	Drug therapy	252	6	61
CQ12	Pruritus	80	6	6
CQ13	Drainage	172	6	18
CQ14	Liver transplantation, indication	78	0	18
CQ15	Outcome, prognosis	210	6	26
CQ16	Population-based, guideline, varices, bone disease, malignancies, gallbladder, cholangiocarcinoma, inflammatory bowel disease, ulcerative colitis	822	6	18

**Table 4:** Items of clinical guidelines: levels of evidence, strength of recommendation and 5-step scale for voting

Level of evidence
(A): Based on strong evidence
(B): Based on moderate evidence
(C): Based on weak evidence
(D): Based on very weak evidence
Strength of recommendation
1: Strongly recommended
2: Weakly recommended (proposed)
None: Cannot make a clear recommendation
Five-step scale for voting
(A) Accept completely
(B) Accept with some revision
(C) Accept with major revision
(D) Reject with revision
(E) Reject completely

The panel of experts evaluated the revised guidelines after face-to-face meeting, strength of recommendations and levels of evidences using the modified Delphi Method based (Table 4). The evaluations were done on the voting on following 5-step scale: (A) accept completely, (B) accept with some revision, (C) accept with major revision, (D) reject with revision, and (E) reject completely (Table 4). The process of debate and revision continued until the combined total of A + B exceeded 80%. The results of voting on acceptance were described on each CQ as level of agreement. This draft of the guidelines that was produced using this method was reviewed by the Scientific Committee of the Japan Biliary Association where it underwent further revisions. Public comments were then requested on the homepage of the Japan Biliary Association. The guidelines were then completed after a final round of debate. We hope that these guidelines will contribute to the improvement and development of the medical care of PSC.

### CQ1. What is the pathophysiology of primary sclerosing cholangitis?


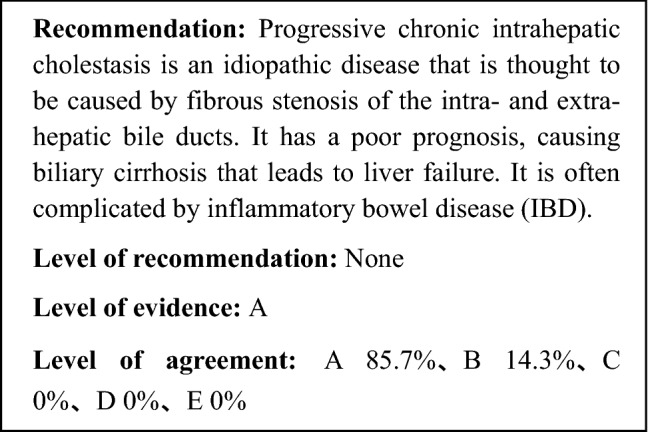


#### Explanation

PSC is an idiopathic progressive chronic intrahepatic cholestasis that is thought to be caused by fibrous stenosis of the intra- and extrahepatic bile ducts. It has a poor prognosis, causing biliary cirrhosis that leads to liver failure. PSC is often complicated by IBD, particularly ulcerative colitis (UC) [1–3]. The etiology and pathophysiology of PSC are thought to be an immunological or genetic abnormality caused by failure of the protective mechanism of the mucosa of the large bowel [4–8]. Specifically, it is thought that PSC complicated with IBD causes continuous inflow of bacteria into the portal vein and mobilization of activated T cells, which in turn causes continuous destruction of the bile duct through the action of MadCAM-1 and CCL25 that have been expressed in hepatic vascular endothelial cells; however, this hypothesis remains unverified [8–11]. Recently, a cholangiopathy concept based on senescence-associated secretory phenotype (SASP) and autophagy that accompanies cell aging has been proposed as a possible pathophysiology for PSC [6, 7, 12–15].

PSC is classified into the following three types based on the location of the bile duct that has been damaged: (1) small duct type, in which there are lesions on the smaller intrahepatic bile duct that cannot be imaged using cholangiography, (2) large duct type, in which the lesions are observed on the larger extrahepatic bile duct, and (3) global duct type, in which damage has occurred in both sites [16]. Recently, sclerosing cholangitis lesions accompanied by autoimmune pancreatitis and sclerosing cholangitis accompanied by IgG4-related disease (IgG4-SC) have been reported as lesions that resemble large duct type PSC. Therefore, care must be taken to differentiate these diseases [16].

**Fig. 1:**
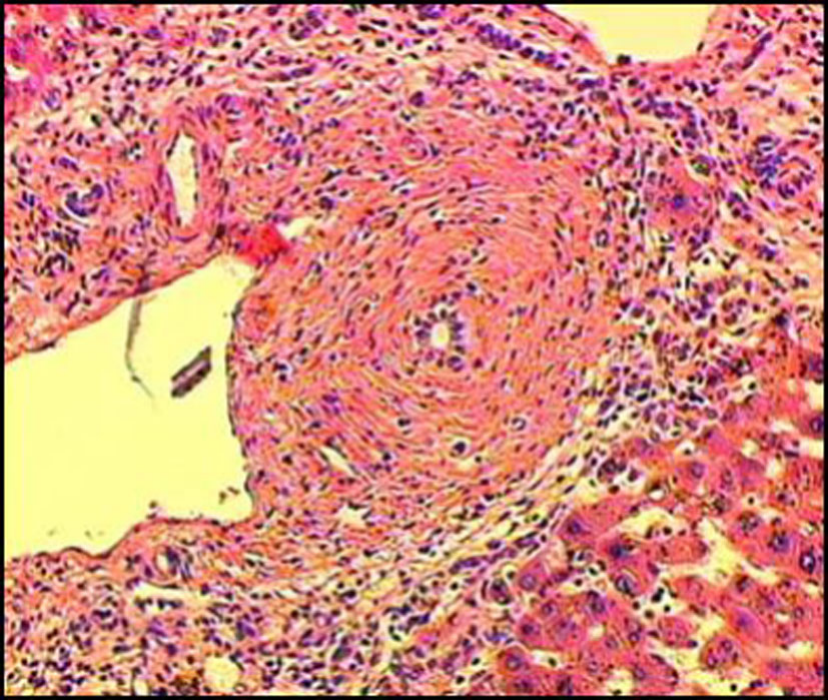
Histopathological findings of the liver indicating PSC (Modified citation from Ref. [18]). Characteristics include ring-like fibrosis surrounding the bile duct and inflammatory cell infiltration. Concentric, nested fibrosis that resembles and onion and is therefore known as “onion-skin fibrosis”—is presented

Histopathologically, PSC is characterized by ring-shaped fibrosis surrounding the bile duct and the infiltration of inflammatory cells. It presents concentric, nested fibrosis that is known as “onion-skin fibrosis” due to its resemblance to an onion (Fig. 1). When the disease concept for PSC was first reported, it was thought that histopathological liver findings from a liver biopsy were necessary, but this is not currently considered to be required; rather, it is considered supplementary to clinical staging [1]. Staging based on histopathological findings (stage 1: cholangitis or portal hepatitis; stage 2: periportal fibrosis or periportal hepatitis; stage 3: septal fibrosis, bridging necrosis, or both; stage 4: biliary cirrhosis) has been proposed. These are to be compared with the clinical pathophysiology.

### CQ 2. What is the epidemiology of primary sclerosing cholangitis?


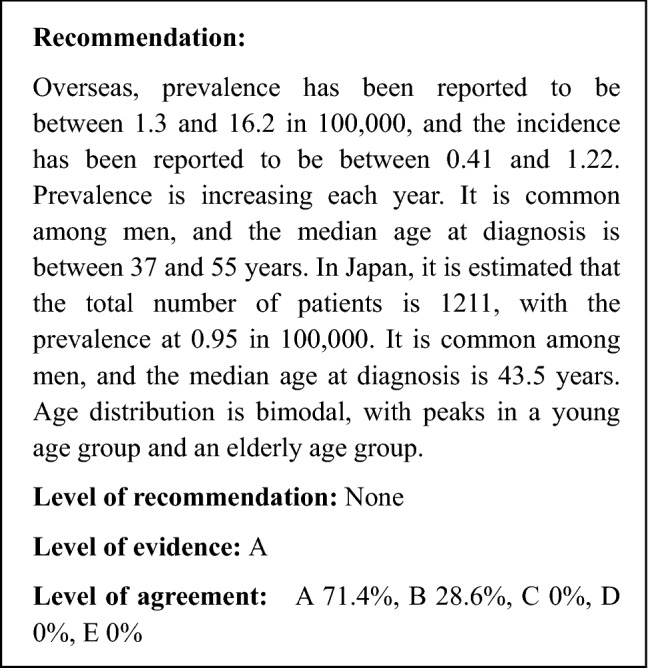


#### Explanation

Most of the high-quality epidemiological studies on PSC have been conducted in Western Europe and North America [17–27]. The only studies that have been conducted in Asia are a 2002 study that was conducted in Singapore [28], as well as a 2007 epidemiological study [29] and National Surveys conducted in 2012 and 2015 [30] in Japan. The results of epidemiological studies conducted in Europe, North America, and Japan up to the present are shown in Table 5. According to these results and meta-studies [31, 32], in Europe and North America the prevalence of PSC is between 3.85 and 16.2 in 100,000 (excluding an older study conducted in Spain in the 1980s), and the incidence is between 0.41 and 1.22. Several epidemiological studies have suggested that the prevalence of PSC is increasing annually [20, 23, 24, 26]. However, it has also been indicated that what appears to be an increase in prevalence in PSC may actually be due to advances in diagnostic technologies, such as ERCP and MRCP, and increases in the prevalence of inflammatory bowel disease, which is a frequent complication of PSC. An epidemiological study conducted in Japan in 2007 reported that the estimated number of PSC patients was 1211 (95% CI 774–1648 people) and that the prevalence was 0.95 in 100,000 (95% CI 0.61–1.28). These figures were considerably lower than those for Western Europe and North America. An epidemiological study conducted in Singapore also reported that the prevalence was low, at just 1.3, which seems to indicate that the prevalence of PSC among Asians is lower than that of Europeans and Americans. However, it remains unknown as to whether the prevalence rate for PSC in Asia has been increasing as it has in the West. Further epidemiological research in this issue is required.

**Table 5:** Epidemiology of primary sclerosing cholangitis

Author	Year of publication	Country/region	No. of PSC patients	Males (%)	Prevalence (95% CI)	Incidence (95% CI)	Age at diagnosis (median, age)
Escorsell et al. [23]	1994	Spain	43	60	0.22	0.07	42.3*
Berdal et al. [18]	1998	Norway	12	58	5.6	0.7	43*
Byron et al. [21]	1996	Canada	39	N/A	6.5	N/A	N/A
Boberg et al. [19]	1998	Norway	17	71	8.5 (2.8–14.2)	1.3 (0.8–2.1)	37
Ang et al. [28]	2002	Singapore	10	90	1.3	N/A	N/A
Bambna et al. [17]	2003	US	22	68	13.6	0.9	40
Kingham et al. [25]	2004	UK	53**	62	12.7	0.91	52
Kaplan et al. [24]	2007	Canada	49	55	N/A	0.92	41
Card et al. [22]	2008	UK	149	63.5	3.85 (3.04–4.80)	0.41 (0.34–0.48)	55
Lindkvist et al. [26]	2010	Sweden	199	71	16.2	1.22	38.5
Toy et al. [27]	2011	US	169	60	4.15	0.41	44.2
Boonstra et al. [20]	2013	Netherlands	590	64	6.0	0.5	38.9*
Reports from Japan [29, 30]	2008, 2016	Japan	435	60	0.95**	N/A	43.5

*PSC* primary sclerosing cholangitis, *N/A* not available

*Mean value

**Prevalence rates are according to the 2008 epidemiological study [29]

In Western Europe, North America, and Japan, PSC is somewhat more common in men. The distribution rate for men is approximately 55–68%. It has been reported that the median age at the time of diagnosis is between 37 and 55 years. National surveys conducted in Japan have also reported the median age at the time of diagnosis as 43.5 years, which is nearly the same as in other regions. However, the age distribution for PSC in Japan is characterized by a bimodal distribution, with peaks in a young age group and an elderly age group. The same trend has recently been reported in Canada [24] and California [27] as well.

### CQ 3. What are the features of primary sclerosing cholangitis in Japan?


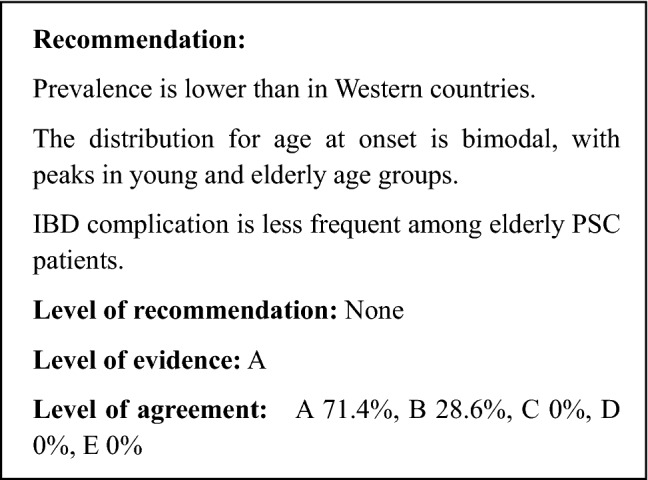


#### Explanation

A review of previously published studies indicates that in Japan, the features of PSC in Japan can be organized into the four areas of epidemiology, complications, pharmacotherapies, and prognosis following liver transplantation.

**Fig. 2:**
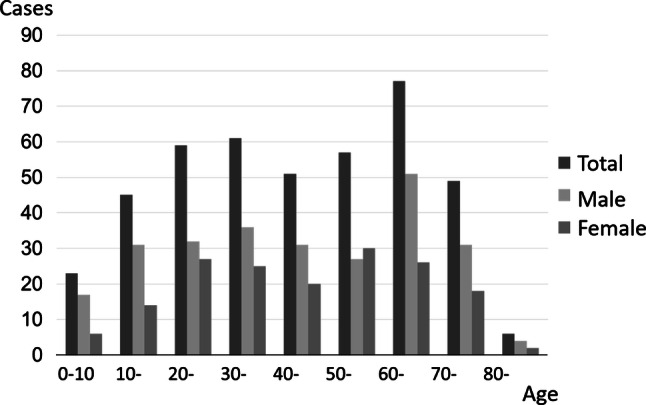
Distribution of age at diagnosis for PSC patients in Japan (Modified citation from Ref. [30])

**Fig. 3:**
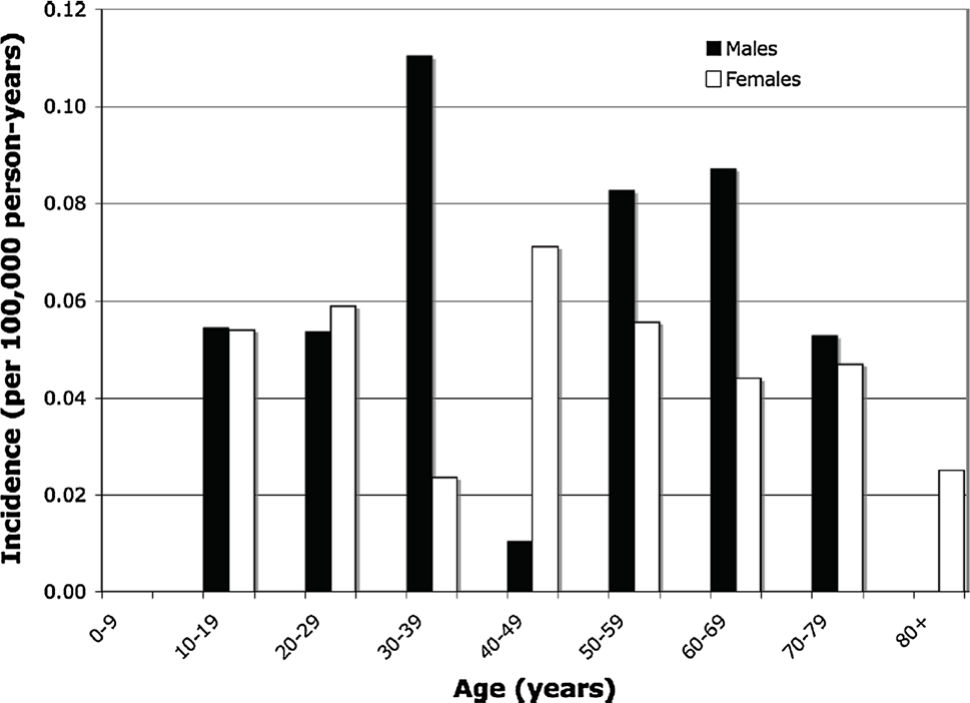
Distribution of age at diagnosis of PSC in Northern California (Modified citation from Ref. [27])

The epidemiological features of PSC in Japan have been studied four times in the past—in 1995 [33], 2003 [34], 2012 [35], and 2015 [30], as well as most recently in 2015, in a study that enrolled 435 subjects. These studies came up with similar results. The Japanese features that differ from epidemiological surveys done in Western countries include the fact that the distribution of ages at onset in Japan was split between the two peaks in young and elderly age groups, and the fact that IBD complication was less frequent in Japan. As shown in Fig. 2, the two age at onset peaks were in the 30s and 60s. This trend was particularly marked among males. This type of bimodal distribution has also recently been reported in Canada [24] and California, USA [27] as well, suggesting that this feature is not unique to Japan (Fig. 3).

Examination of complications indicated that in the 1995 and 2003 surveys autoimmune pancreatitis (AIP) was common among elderly PSC patients in both years. However, IgG4-related sclerosing cholangitis (IgG4-SC) was also included with AIP as a kind of IgG4-related disease [36]. Thus, when IgG4-SC was excluded from the start in the surveys conducted in 2012 and 2015, results indicated that there were no AIP or pancreatic lesion complications. According to epidemiological surveys conducted in Western countries, 60–80% of PSC patients experience complications with inflammatory bowel diseases (IBD), which is less common in Japan, where the total rate in the 2015 survey was 39.5%. However, when the total number of PSC patients is divided by age groups, the rate of IBD complication is 60.9% among the younger patients and 18.3% among the elderly patients, indicating that the IBD complication rate among younger patients is on par with that in Western countries, while that of elderly patients is extremely low. In other words, the data suggest the possibility that PSC among elderly Japanese patients has a unique feature that is not seen among Western elderly PSC patients [37]. Although it has been reported that the prognosis for elderly onset PSC is better than that for early onset PSC [38], the 2015 survey indicated that the prognosis for elderly onset PSC was poor [30]. Further investigation of this, as well as investigation of whether elderly onset PSC should in fact be considered “primary” sclerosing cholangitis, is required going forward.

### CQ 4. What are the risk factors for primary sclerosing cholangitis?


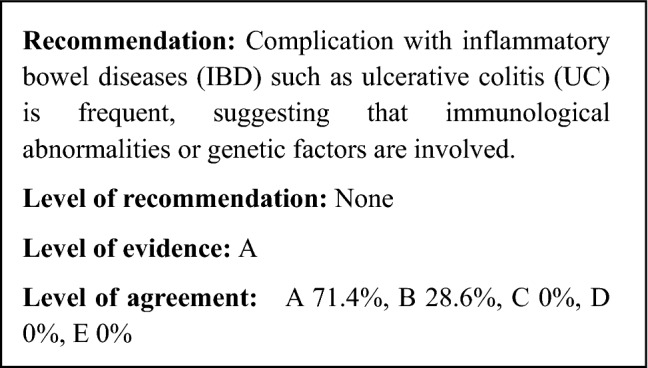


#### Explanation

Early onset PSC is commonly complicated by IBD, and especially ulcerative colitis (UC), with a particularly high complication rate observed among males [34, 39]. Cited reasons for such a large number of PSC cases being complicated with UC include the fact that there is active inflammation in the intestinal tracts of UC patients, permeability of the intestinal mucosa is promoted, the intestinal microbiota and bacterial components are related to the onset of PSC, and absorption of bile acid produced by the intestinal tract leads to liver and bile duct damage [40, 41].

PSC is also known to run in families. Those with siblings or other first-degree relatives who have PSC are more likely to develop PSC than the general population, and are more likely to develop IBD [40, 42]. Human leukocyte antigen (HLA)-B8, B27, HLA-DRW52, and other HLA haplotypes are known to be involved in PSC onset [43]. Investigations using genome-wide association studies (GWAS) have identified disease susceptibility genes in 16 locations [44–47]. This suggests that many of these share a common onset route with other autoimmune diseases.

### CQ 5. What are the symptoms that suggest primary sclerosing cholangitis?


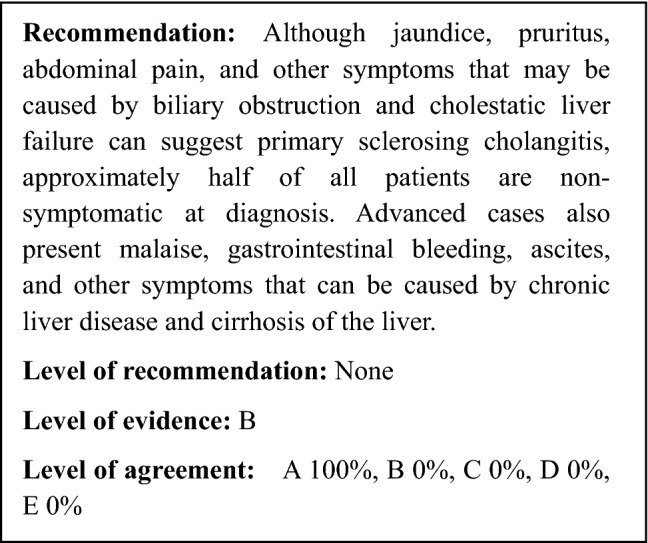


#### Explanation

Cases of primary sclerosing cholangitis present symptoms that can be broadly divided into three types of pathophysiologies. These are symptoms that are caused by biliary obstruction and cholestatic liver failure, symptoms that are due to advanced chronic liver disease and cirrhosis of the liver, and symptoms that are caused by inflammatory bowel diseases that complicate PSC. However, symptoms that are caused by IBD are naturally not symptoms of PSC. Symptoms caused by cholestasis include jaundice, cholangitis, pruritus, and abdominal pain, but jaundice and pruritus are also presented in cases of advanced liver failure, which makes differentiation by symptoms alone difficult. While there have been reports of gastrointestinal bleeding from varix rupture and ascites in cases of liver cirrhosis, opportunities for diagnosing PSC by such symptoms in Japan have become rare in recent years. Malaise is a non-specific symptom that is thought to occur in conjunction with chronic liver disease and jaundice. The American College of Gastroenterology’s Clinical Guideline also lists malaise, pruritus, jaundice, and gastrointestinal bleeding among the symptoms of PSC [48]. A study that compared PSC around the world reported that although PSC in Japan was infrequently complicated with IBD, there were no differences between Japan and other countries regarding general symptoms [49]. A national survey conducted in Japan in 2014 reported on PSC cases diagnosed since 2005 [35]. Of a total of 197 cases, 100 were asymptomatic (55%), 46 suffered jaundice (25%), 37 suffered cholangitis (20%), and 31 suffered pruritus (17%). Since these symptoms were identified during analysis and not at the time of diagnosis, if one considers the possibility that at least some of these symptoms appeared during the course of the disease, the number of cases that were asymptomatic at the time of diagnosis would increase. Table 6 shows the data related to PSC symptoms reported in countries around the world [23, 26, 28, 50–53]. As these were extracted from cohort studies published in each country and, therefore, range over a long period of time, the older studies may include cases of IgG4-SC. Many recent studies report that, as in studies conducted in Japan, approximately half of the cases were asymptomatic. It is likely that these cases were discovered as a result of follow-up testing for cholestatic liver failure that was first identified during medical checks and other types of regular medical examinations. Cases in which IBD was discovered during the course of the disease were likely to have been discovered as a result of liver disease prior to becoming symptomatic.

**Table 6:** Reported symptoms of primary sclerosing cholangitis

Author	Country	Year	Cases	Male (%)	Age* (years)	IBD (%)	Asymptom (%)	Jaundice (%)	Cholangitis (%)	Pruritis (%)	GI bleeding (%)	Ascites (%)	Fatigue (%)	Abd. pain (%)
Tanaka et al. [35]	Japan	2014	197	53.8	48.1	34.0	55.0	25.0	20.0	17.0	N/A	N/A	N/A	N/A
Garioud et al. [50]	France	2010	150	63.3	44.0	60.0	48.0	21.0	13.0	22.0	7.0	8.0	36.0	N/A
Lindkvist et al. [26]	Sweden	2010	199	71.4	38.5	76.4	53.3	24.1	9.5	10.1	N/A	N/A	N/A	25.1
Ataseven et al. [51]	Turkey	2009	35	60.0	41.7	62.9	31.5	20.0	N/A	31.4	N/A	N/A	8.6	8.6
Ang et al. [28]	Singapore	2002	10	90.0	50.9	20.0	20.0	10.0	70.0	0.0	N/A	N/A	N/A	N/A
Kochhar et al. [52]	India	1996	18	61.1	39.0	50.0	16.7	83.3	16.7	N/A	27.8	N/A	N/A	N/A
Broome et al. [53]	Sweden	1996	305	64.0	39.0	91.0	44.0	30.0	17.0	30.0	4.0	4.0	N/A	37.0
Escorsell et al. [23]	Spain	1994	43	60.0	42.3	46.0	16.0	69.0	28.0	59.0	N/A	N/A	69.0	N/A

*IBD* inflammatory bowel disease, *GI bleeding* gastrointestinal bleeding, *Abd. pain* abdominal pain, *N/A* not available

*Mean

### CQ 6. What are the characteristic blood test findings for primary sclerosing cholangitis?


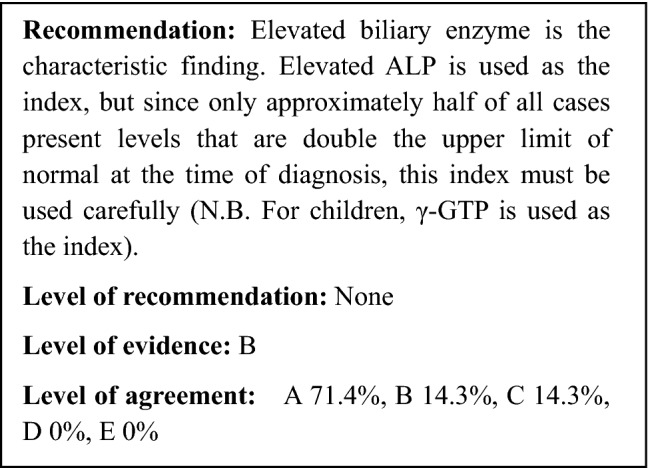


#### Explanation

Primary sclerosing cholangitis is a chronic liver disease that causes multiple, diffuse strictures of the intra- and extrahepatic bile ducts, which leads to cholestasis. Diagnosis of PSC has generally been performed using the 2003 Mayo Clinic diagnostic guidelines [1]. According to these guidelines, diagnosis is indicated when serum ALP levels are maintained at two to three times normal levels for at least six continuous months. Although significantly elevated ALP level is the main index used to indicate cholestasis, some cases present normal levels, and it has been reported that γ-GTP shows gradual elevations in some cases [48, 54–56]. ALP level data at the time of diagnosis in Japan show that although only 215 cases (54.2%) had levels elevated to above two times the upper limit of normal, 114 cases (28.7%) had levels that were elevated above the upper limit of normal but had not yet reached two time this upper limit, and 68 cases (17.1%) presented levels that were not in excess of the upper limit of normal [57].

In many cases, transaminase levels are elevated to two to three times the upper limit of normal, but as with ALP levels, some cases also present normal levels. In 70% of PSC cases, bilirubin levels are normal at the time of diagnosis, but as symptoms progress the patient will eventually present abnormal levels. In 60% of PSC cases, IgG levels show gradual elevations of 1.5 times the upper limit of normal, but in 9% of cases IgG4 shows only slight elevations [48, 54, 55]. In Europe and the US, elevated IgM levels are presented in 50% of cases [48]. In Japan, 24% of cases exceed the IgM cutoff level of 200 mg/dl, and it has been reported that younger PSC patients tend to present high IgM levels [38, 58, 59].

Overlap of PSC and autoimmune hepatitis (AIH) occurs in approximately 10% of cases (1.4–17%), and it is common in young adult and pediatric PSC patients [48, 60, 61]. Findings such as transaminase level elevations of five times or more the upper limit of normal and autoantibody-positive (antinuclear antibody, smooth muscle antibody, etc.) suggest AIH. Since ALP levels increase with the bone development in pediatric PSC patients, it is recommended that γ-GTP was used to confirm cholestasis [48, 54].

A variety of autoantibodies have been reported in cases of PSC [48, 54, 55, 62]. Antinuclear antibodies are detected in 7–77% of cases, perinuclear anti-neutrophil cytoplasmic antibody (p-ANCA) is detected in 50–80% of cases, smooth muscle antibodies are detected in 13–20% of cases, intrinsic factor antibodies are detected in 35% of cases, anticardiolipin antibodies are detected in 4–66% of cases, thyroid peroxidase antibodies are detected in 7–16% of cases, thyroglobulin is detected in 4% of cases, and rheumatoid factor is detected in 15% of cases. However, in all such cases, the levels are low. As these antibodies are not specific to PSC, they are not recommended for use as a screening test for PSC diagnosis. In Japan, 37.3% of cases are antinuclear antibody-positive and 2.4% are p-ANCA-positive. The positive rate for p-ANCA in Japan has been found to be lower than those reported in Western countries [63].

### CQ 7. What are the diagnostically useful imaging findings for primary sclerosing cholangitis?


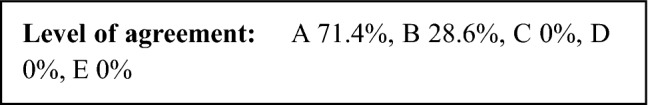


#### Explanation

**Fig. 4:**
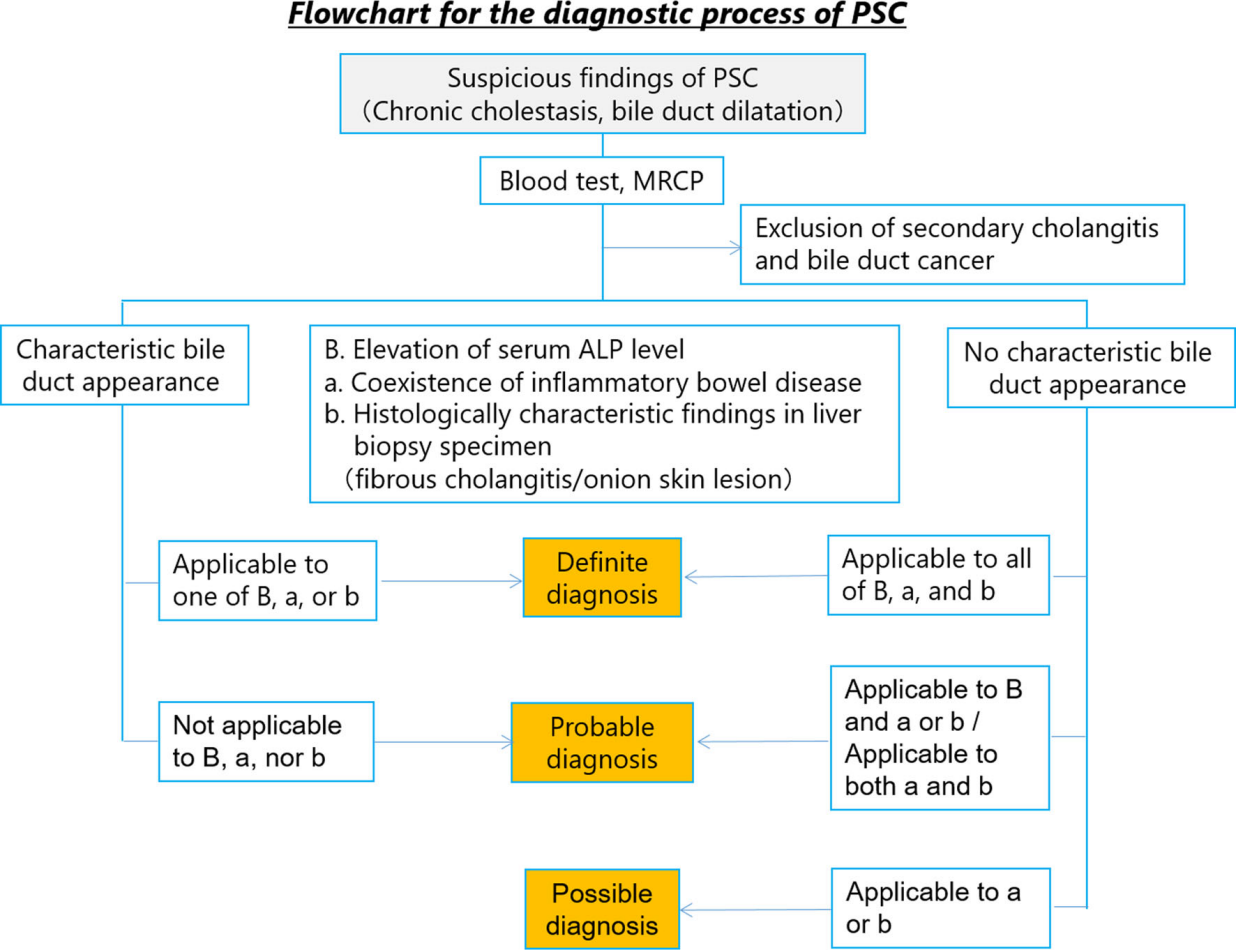
Flowchart of diagnostic strategies of PSC

In this guideline, PSC was diagnosed based on the 2016 diagnostic criteria for PSC which was published by same group previously [64]. We had made flow chart for diagnostic strategies of PSC according to these diagnosis criteria (Fig. 4). This flow chart was combined with symptoms, image findings and blood tests, and differential diagnosis of secondary-SC was also important point of this flow chart.

### CQ7-1. What are the first-line imaging examinations?


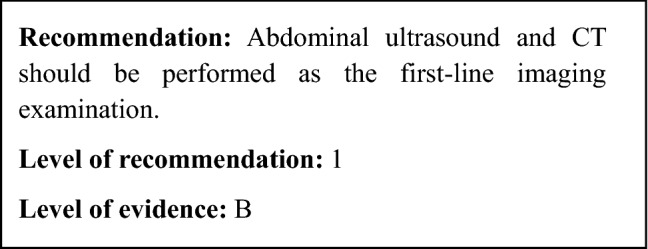


#### Explanation

**Fig. 5:**
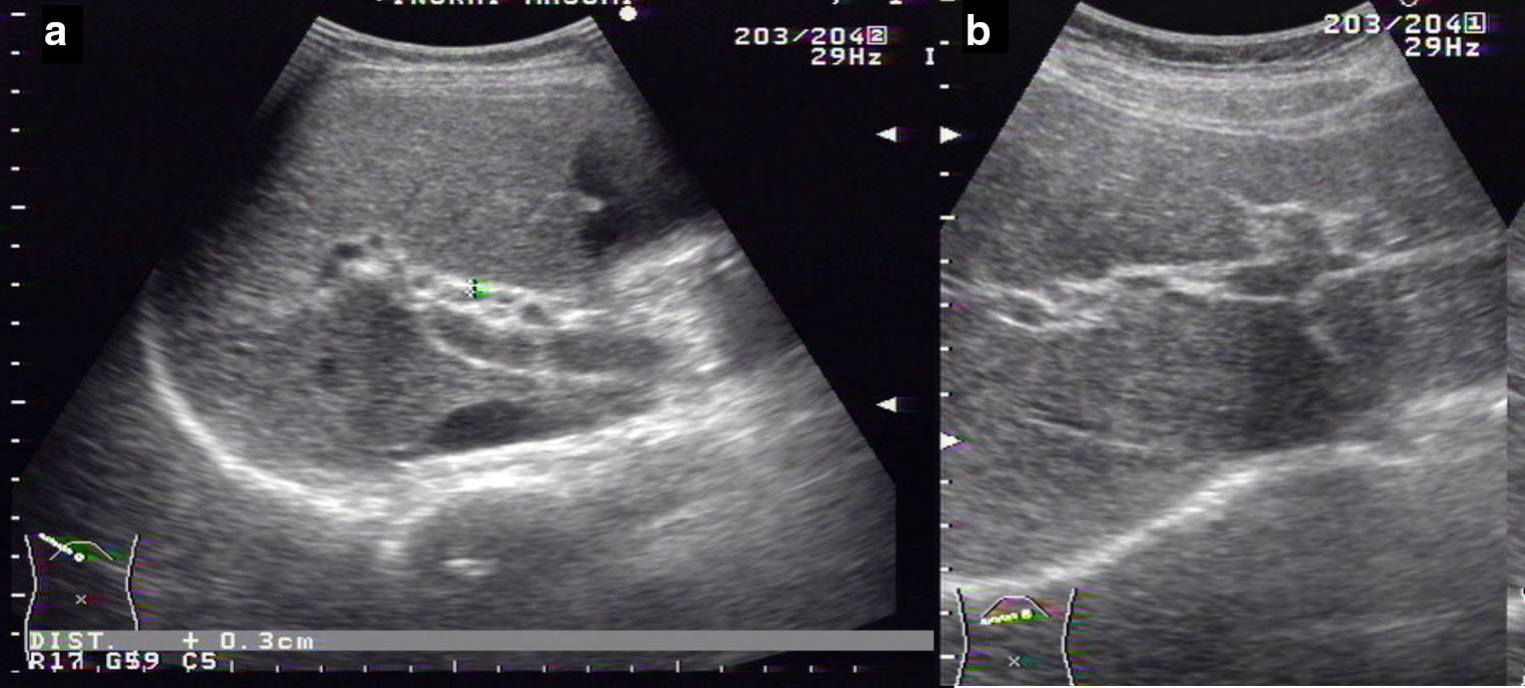
Ultrasound Image of PSC. **a** Common bile duct wall thickening is observed. (right intercostal scanning). **b** Intrahepatic bile duct at the umbilical region are dilated and have thickened irregularly. (wide probe scanning)

When blood chemistry analysis indicates stasis, abdominal ultrasound and abdominal CT are performed to differentiate the condition from obstructive jaundice [48]. Dilation of the extrahepatic bile duct, thickening of the bile duct walls, or gallbladder enlargement [65, 66] are findings that suggest sclerosing cholangitis (Fig. 5). To diagnose PSC, it is necessary to exclude secondary sclerosing cholangitis and IgG4-related sclerosing cholangitis. IgG4-related sclerosing cholangitis is usually complicated with autoimmune pancreatitis. Thus, it is necessary to investigate pancreas-related findings in order to determine whether the case is complicated by IgG4-related diseases such as sclerosing sialadenitis, pulmonary lesions, or retroperitoneal fibrosis [67].

### CQ7-2. What are the second-line imaging examinations?


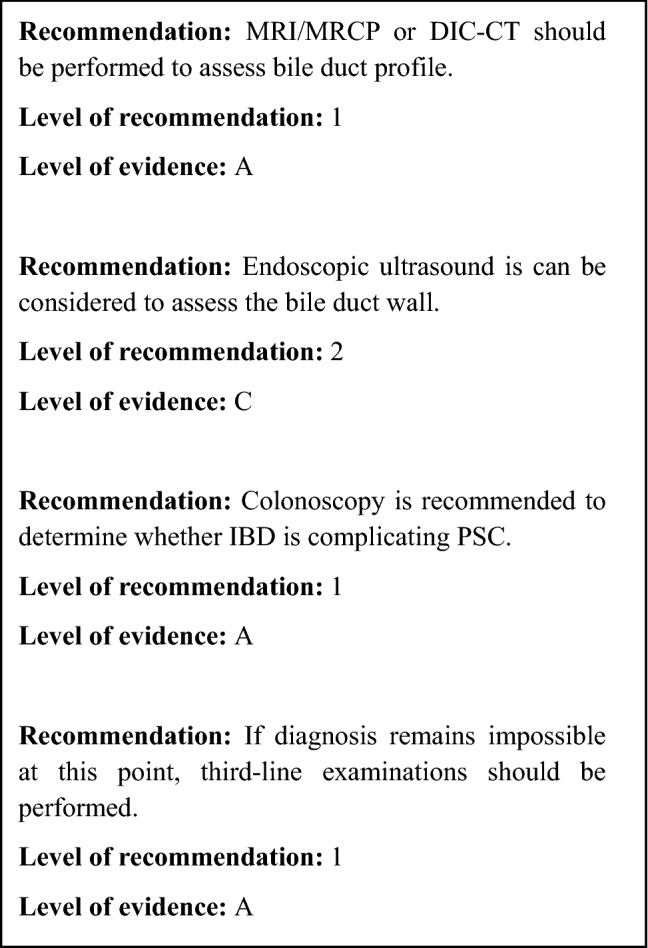


#### Explanation

**Fig. 6:**
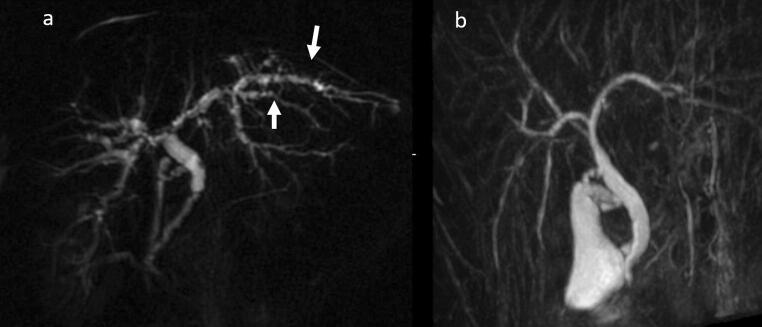
MRCP Image of PSC. **a** Alternations between strictures and slightly dilated segments produce a beaded pattern. (white arrow). **b** This image shows non-stricture of the common bile duct in the part of the duodenal side from the cystic duct, it also demonstrates gallbladder enlargement. This enlargement is non-specific but refers to findings of PSC

PSC can be diagnosed when MRCP and DIC-CT (drip infusion cholecystocholangiography-CT) indicate findings characteristic of PSC such as beaded appearance (Fig. 6). MRCP should be performed first because the precision of MRCP has improved, it is less invasive than ERCP and can be performed at a low cost (see CQ. 8) [68–77].

Endoscopic ultrasound will show that the bile duct wall thickening is diffuse. Study of thickening of the common bile duct walls indicated 0.8 ± 0.4 mm as the normal control, and 2.5 ± 0.8 mm for PSC as opposed to 0.8 ± 0.4 mm for choledocholith [78]. There are cases in which characteristics indicated using intraductal ultrasonography, which are explained in CQ. 7-3, are also indicated by endoscopic ultrasound.

**Fig. 7:**
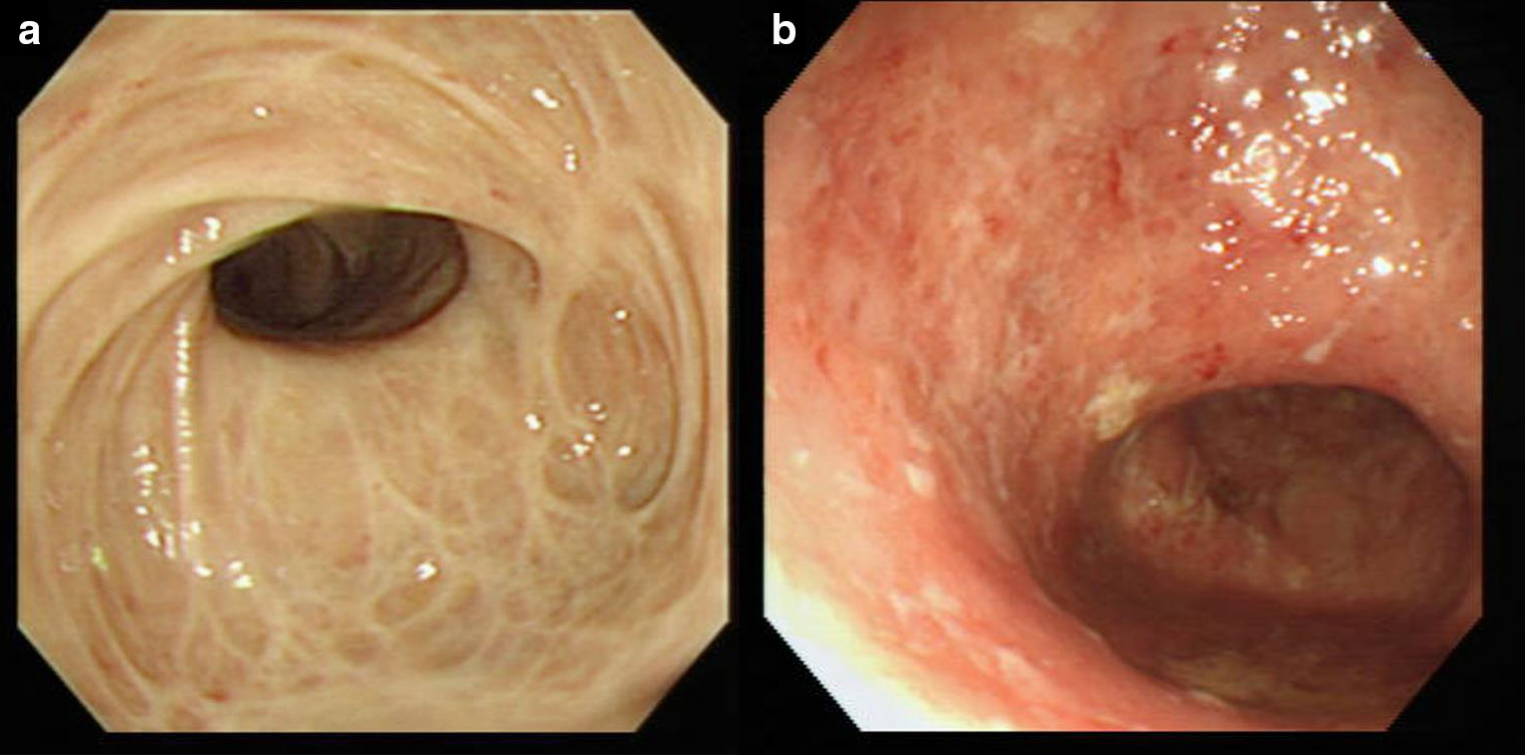
Colonoscopy image of the inflammatory bowel disease accompanied with PSC. **a** This picture shows multiple ulcerative stenosis in the area from the ileocecal to the ascending colon, but no rectal lesion has been found. (at the time of this diagnosis). **b** Multiple erosion and vascular permeability reduction have appeared in the same area (7 months after the diagnosis)

Since PSC is frequently complicated with IBD, colonoscopy should be performed even in the absence of any symptoms. IBD complication is particularly common in cases of early onset PSC [35, 59]. Since PSC is characterized by lack of rectal lesions and intense inflammation of the right colon unlike normal cases of ulcerative colitis, these findings can be useful in diagnosing PSC (Fig. 7) [79–83].

### CQ 7-3. What are the third-line imaging examinations?


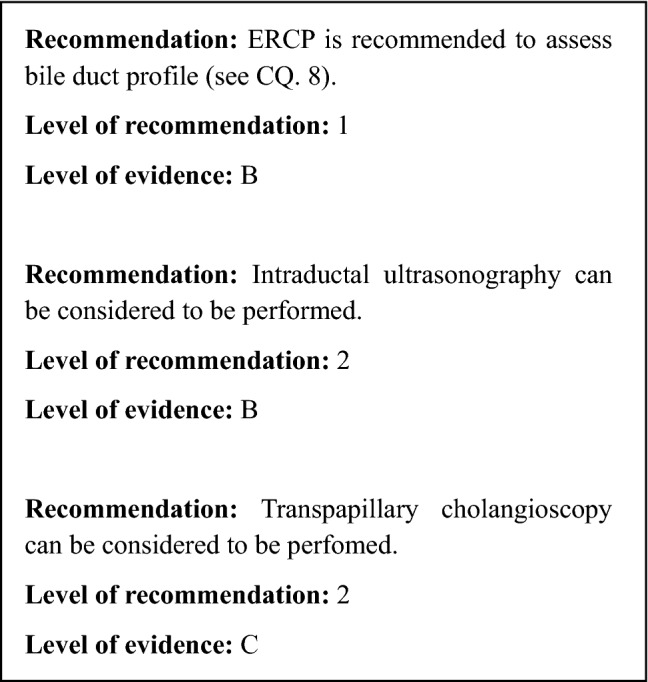


#### Explanation

Since ERCP entails the risk of procedural accidents such as post-ERCP pancreatitis and exacerbation of cholangitis [73], it is often performed in cases in which less invasive examinations such as MRCP and DIC-CT have failed to reveal characteristic findings of PSC [84–86]. In cases in which malignant diseases should be excluded and there is stricture suggesting complication with cholangiocarcinoma, ERCP is sometimes performed in order to conduct brush cytology or biopsy [87], and in cases in which the patient has experienced repeated cholangitis, it is performed to conduct balloon dilation of the stricture site (see CQ. 13) [88, 89].

**Fig. 8:**
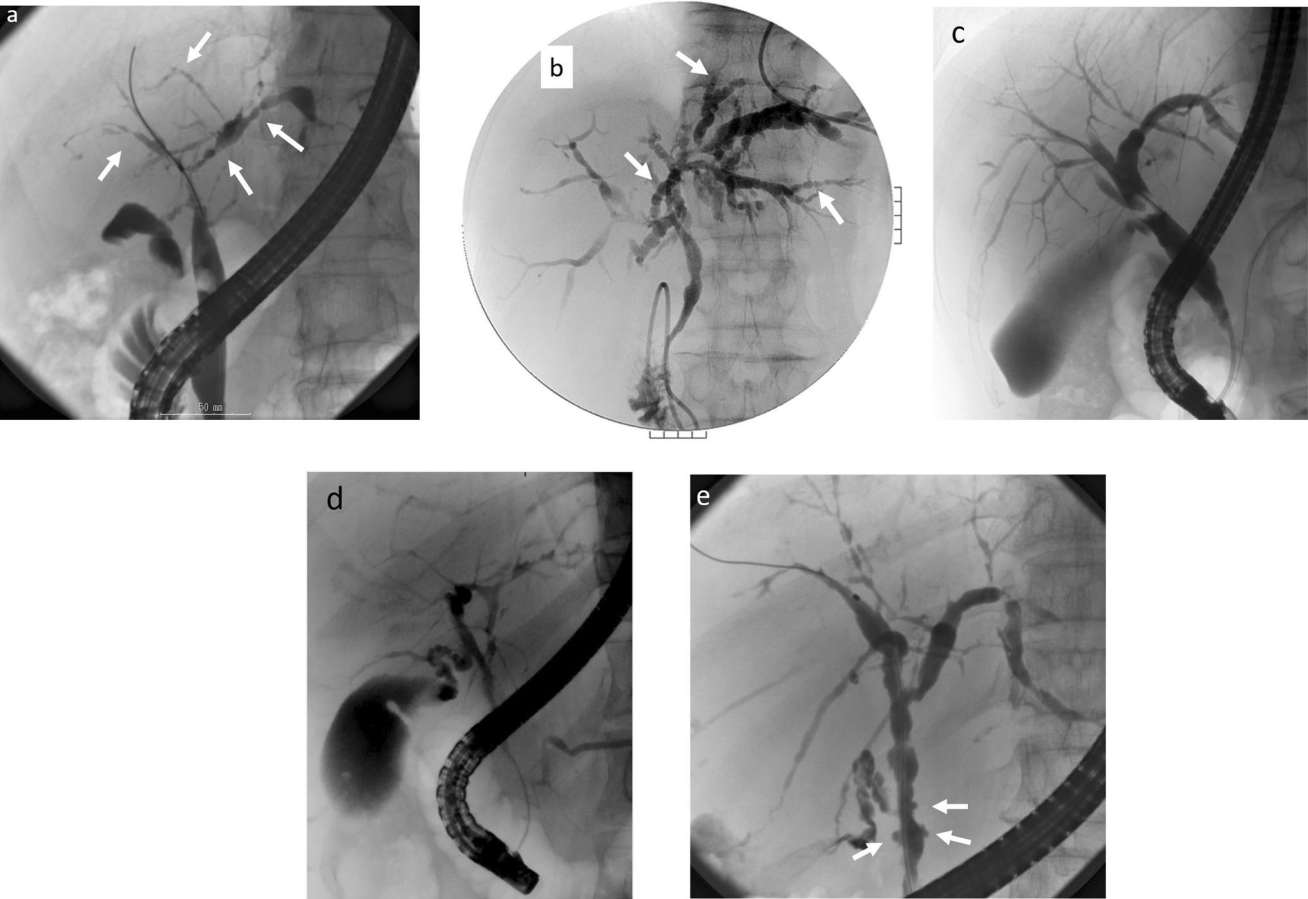
Characteristic bile duct image of PSC. **a** Band-like stricture findings with ERC. Multifocal, short, annular strictures can be observed in the intrahepatic ducts (white arrows). **b** Beaded appearance findings with ERC. Alternations between strictures and slightly dilated segments produce a beaded pattern (white arrows). **c** Pruned tree appearance findings with ERC: Early stage. Overall intrahepatic branch ducts are narrow but can be detected with ERC. **d** Pruned tree appearance findings with ERC: advanced stage. Branch ducts seem to have disappeared as if they were pruned trees. **e** Diverticulum-like outpouching findings with ERC. Extrahepatic bile ducts have outpouching diverticular-like appearance (white arrow)

**Fig. 9:**
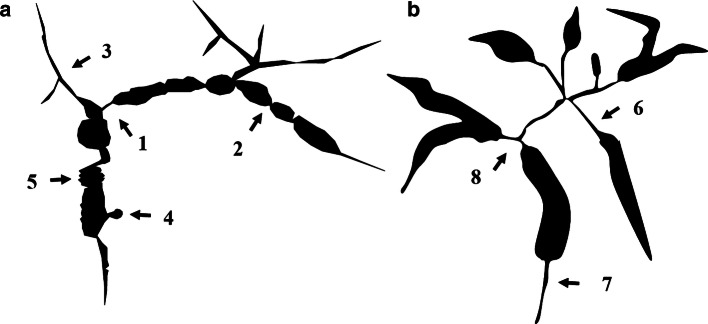
Comparison Between PSC and IgG4-SC with the bile duct image. (Modified citation from Ref. [89]). **a** Primary sclerosing cholangitis. (1) Band-like stricture, (2) beaded appearance, (3) pruned tree appearance, (4) diverticulum-like outpouching, (5) shaggy appearance. **b** IgG4-related SC. (6) Dilation after confluent stricture, (7) stricture of lower common bile duct, (8) stricture of hepatic hilar

ERCP images that are characteristic of PSC include band-like stricture (Fig. 8a), beaded appearance (Fig. 8b), pruned tree appearance (Fig. 8c, d), and diverticulum-like outpouching (Fig. 8e) [90, 91]. In contrast to IgG4-related sclerosing cholangitis, which has a relatively long section of stricture, the stricture is short in PSC (Fig. 9) [90–94].

**Fig. 10:**
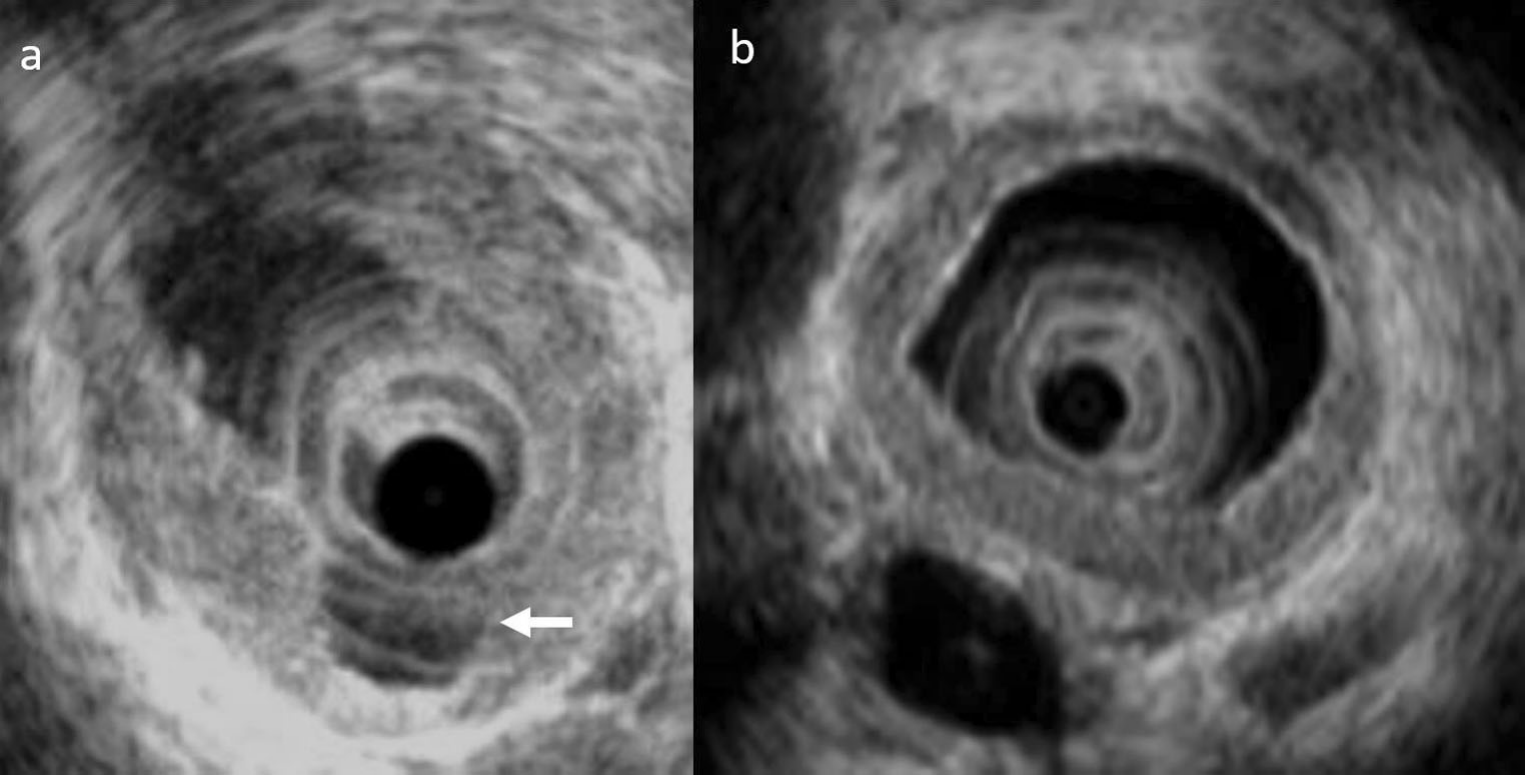
Intraductal ultrasound (IDUS) findings. **a** IDUS of PSC: this image shows an irregularity of the bile duct epithelium and diverticulum-like outpouching (white arrow). **b** IDUS of IgG4-related SC: bile duct epithelium is smooth and preserves three-layer structure. Regular hypoechoic thickening in the inner part can be seen

**Fig. 11:**
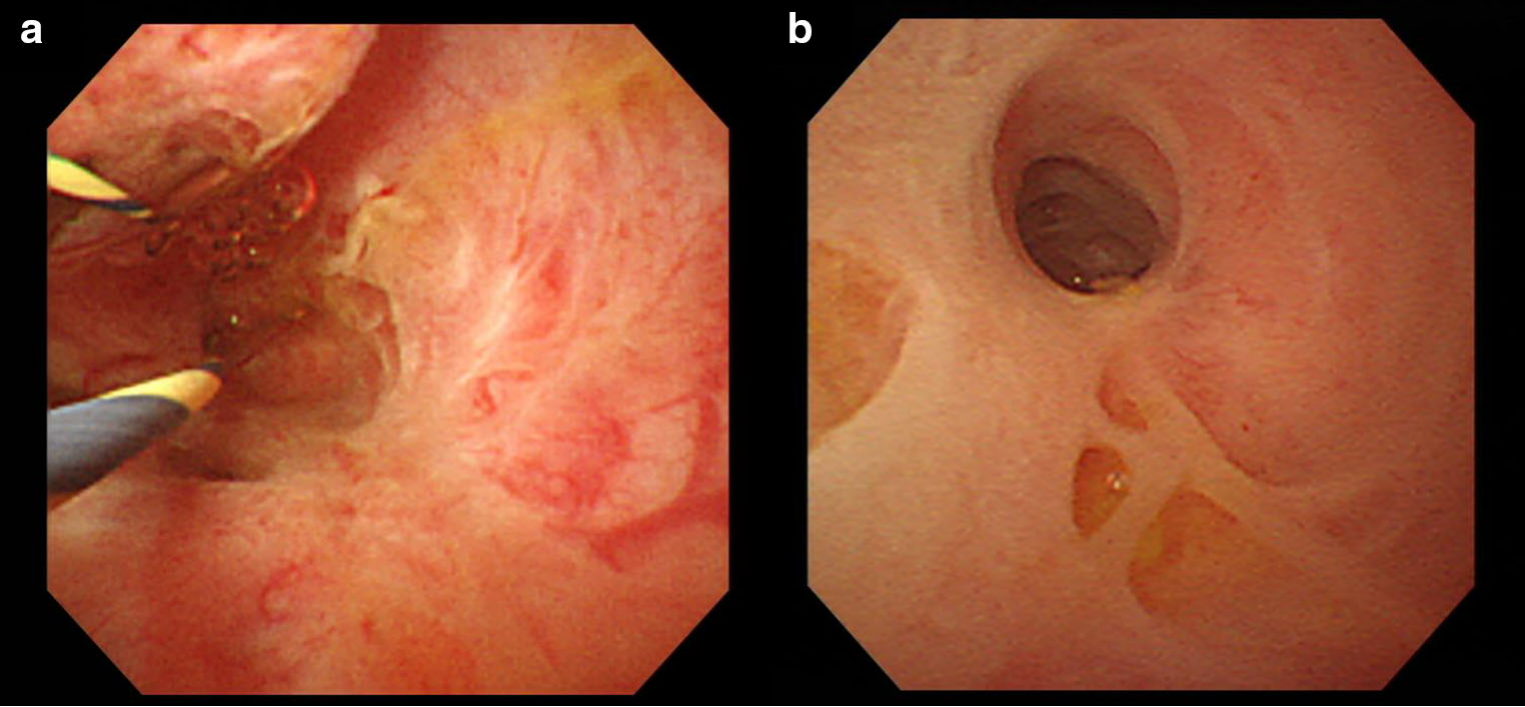
Peroral cholangioscopy (POCS) findings of PSC. **a** Ulcerative scars. **b** Multiple diverticulum-like findings

Intraductal ultrasonography indicates damaged and irregular epithelium in cases of PSC and diverticulum-like outpouching is very common (Fig. 10a) [95, 96]. In cases of IgG4-related sclerosing cholangitis, however, the epithelium remains smooth and is intact through three layers, with the medial hypoechoic layer characteristically showing thickening (Fig. 10b). These findings are contradistinctive to PSC. Transpapillary cholangioscopy characteristically reveals multiple ulcer scars and false diverticula (Fig. 11a, b) [97, 98].

### CQ 8. On what type of cases is endoscopic retrograde cholangiopancreatography (ERCP) performed?


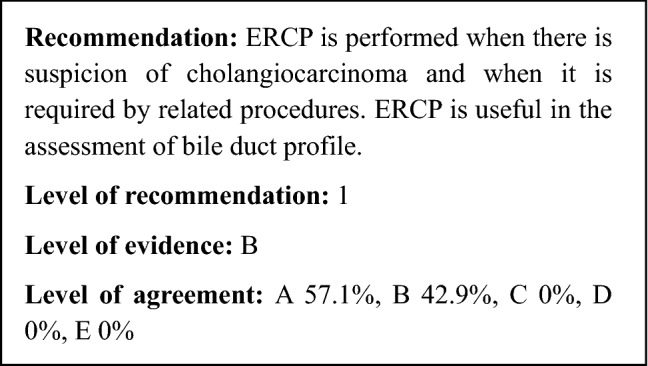


#### Explanation

Primary sclerosing cholangitis (PSC) often causes multiple strictures in the intrahepatic bile duct, and bile duct profile findings characteristic of PSC are required for a diagnosis [48, 54, 55]. Other diseases that also cause bile duct strictures and, therefore, require differentiation from PSC include cholangiocarcinoma, IgG4-related sclerosing cholangitis (IgG4-SC), and secondary sclerosing cholangitis. The characteristic bile duct findings that indicate PSC when using endoscopic retrograde cholangiopancreatography (ERCP) include band-like stricture, beaded appearance, pruned tree appearance, and diverticulum-like outpouching. An extremely detailed reading of ERCP bile duct images is required to differentiate PSC from IgG4-SC [90]. ERCP has long been used to assess bile duct profile, and its usefulness as a diagnostic tool for PSC has made it the standard diagnostic tool [48, 54, 55].

However, as recent improvements in the image resolution of magnetic resonance cholangiopancreatography (MRCP) have made it possible to use this technology to assess bile duct profile, it is now considered a useful tool in the diagnosis of PSC [75–77]. Research that compared the capabilities of ERCP and MRCP to diagnose PSC indicated that ERCP was superior in the imaging of the bile duct branches, but that MRCP was superior in imaging upstream obstructions of the bile duct [75]. Research that compared the diagnostic accuracy of ERCP and MRCP for PSC indicated that both examinations were equal, with the diagnostic sensitivity to PSC of MRCP at 80% or higher and the specificity at 87% or higher [75–77]. It has also been reported that MRCP is less costly to use [76]. Problems with ERCP include the risk of post-ERCP pancreatitis and cholangitis. Pancreatitis after ERCP performed for the purpose of PSC diagnosis occurs in 1.2–7% of all cases and cholangitis occurs in 1.4–2% of all cases [73, 99, 100]. Thus, in recent years MRCP has come to be recommended for the assessment of bile duct profile for the purpose of diagnosing PSC, and it is now in wide use for that purpose [75–77].

However, it is difficult to use MRCP to assess non-severe bile duct profiles such as those in early stage PSC. As a result, when sufficiently clear images of early stage cases of PSC cannot be obtained using MRCP, ERCP is used even now as a useful alternative [101].

In some cases, it is difficult to differentiate between PSC and either cholangiocarcinoma or IgG4-SC with bile duct images alone. Therefore, in addition to ERCP, differential diagnosis can also be performed using endoscopic bile duct brush cytology, biopsy, and bile duct intraductal ultrasonography [96]. PSC is sometimes complicated with choledocholithiasis and cholangiocarcinoma, and the dominant stricture can also cause obstructive jaundice. Thus, endoscopic therapy is useful for procedures such as choledocholithotomy, balloon-assisted dilation of the stricture, and stent placement.

Although ERCP is useful in the assessment of bile duct profile, MRCP is often used to diagnose PSC. ERCP is utilized for differential diagnosis in cases suspected of cholangiocarcinoma as well as in cases that require ERCP-related procedures.

### CQ 9. Is liver biopsy useful for the diagnosis of primary sclerosing cholangitis?


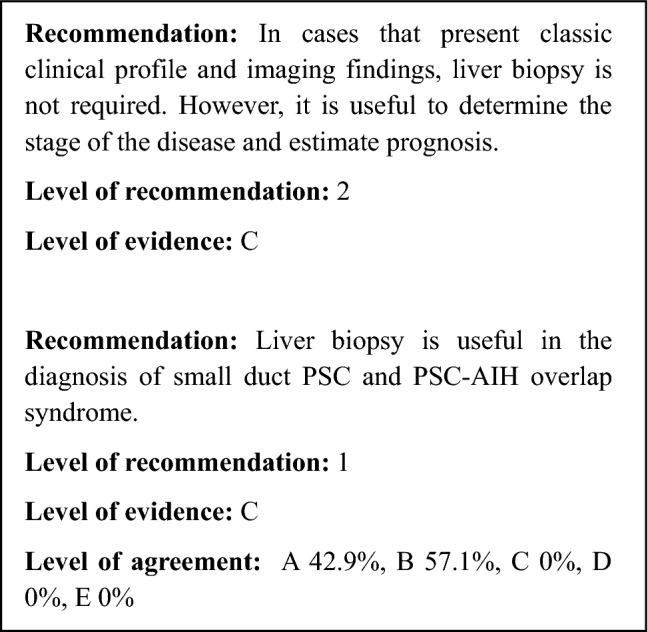


#### Explanation

**Fig. 12:**
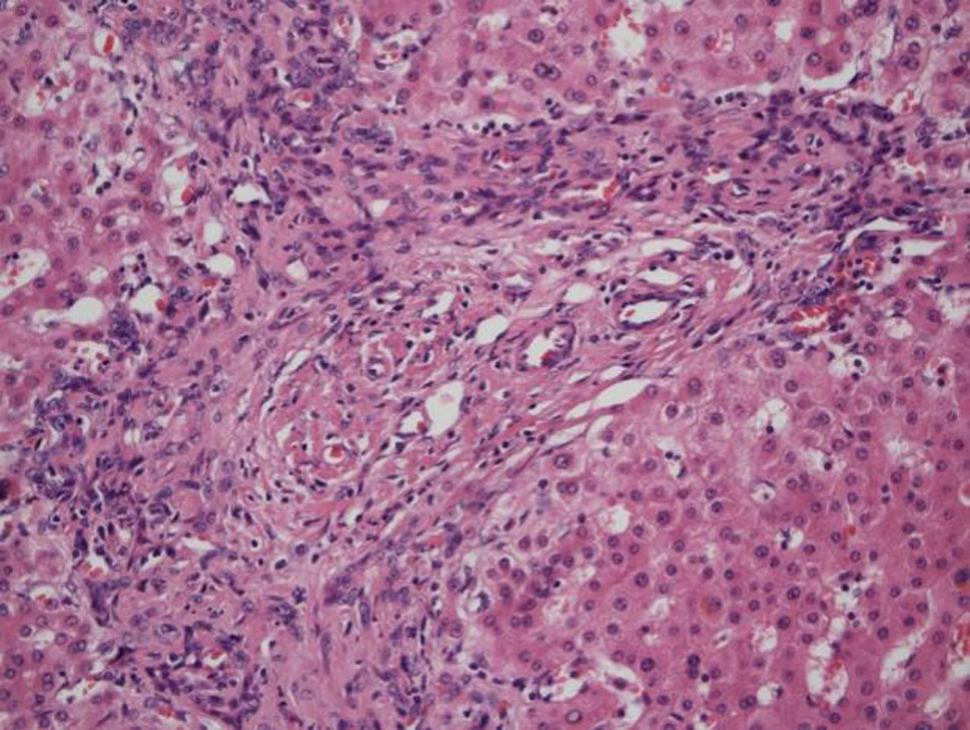
Small duct disappearance and periportal ductular proliferation

The characteristic bile duct lesions in liver biopsies of PSC consist of concentric fibrosis around the bile duct epithelium and stricture of the bile duct lumen, which is known as onion-skin fibrosis (fibrous obliterative cholangitis) [102, 103]. This finding is not necessarily specific to PSC and can be found, for example, in pediatric vanishing bile duct syndrome [103]. In addition, the low frequency (7–50%) to encounter onion-skin fibrosis is another problem of the liver biopsy [102–105]. Liver biopsies for PSC often reveal findings such as degeneration, reduction, and disappearance of the interlobular bile ducts, ductular proliferation (Fig. 12), portal inflammation with fibrosis, and piecemeal necrosis [102, 104, 105], all of which are not considered specific to PSC. A study that reviewed 138 cases of PSC and examined the usefulness of biopsies performed on 79 cases [106] demonstrated that the biopsy diagnosis led to a change of therapeutic strategy in one case only (diagnosed with PSC-AIH overlap syndrome). No changes were made in any of the other cases, and a complication was found in only one case. Based on this study, the EASL [55], AASLD [54], and ACG [48] guidelines indicate that in cases that present the classic clinical profile and imaging findings, liver biopsy is not required. However, liver biopsy is necessary [48, 54, 55] for the diagnosis of PSC-AIH overlap syndrome and small duct PSC [107, 108].

In terms of differentiation between PSC and IgG4-SC using liver biopsy, the presence of numerous IgG4-positive cells and the absence of advanced fibrosis corresponding to stages 3/4 and onion-skin fibrosis have been reported in cases of IgG4-SC [91, 109]. It has also been found in cases of PSC that inflammatory cell infiltration is scarce and perivenous inflammation and inflammatory mass formation are absent [110].

Staging through the use of liver biopsy has been reported to be useful in estimating the prognosis of PSC patients [111]. Using the Ishak, Nakanuma, or Ludwig staging systems, a relationship between the disease stages correlates well with the survival period without transplantation or period until liver transplantation [112]. However, when two biopsied tissue samples taken from a single PSC patient are compared, the stages are discrepant in 27% of cases, suggesting that different findings in different sites can be a problem [113].

### CQ 10. How are differentiation from cholangiocarcinoma and diagnosis of complications performed?


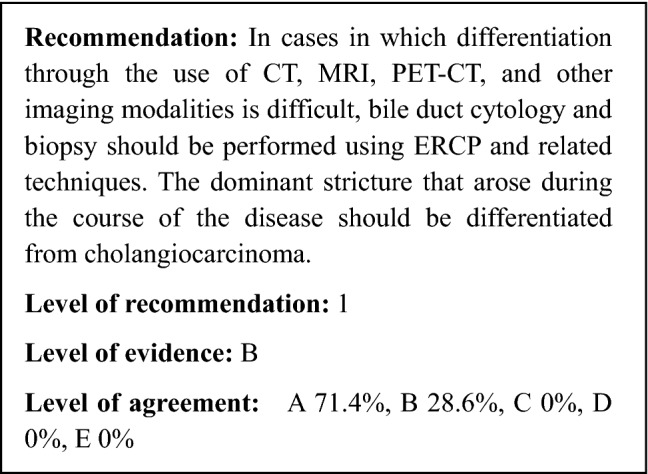


#### Explanation

**Fig. 13:**
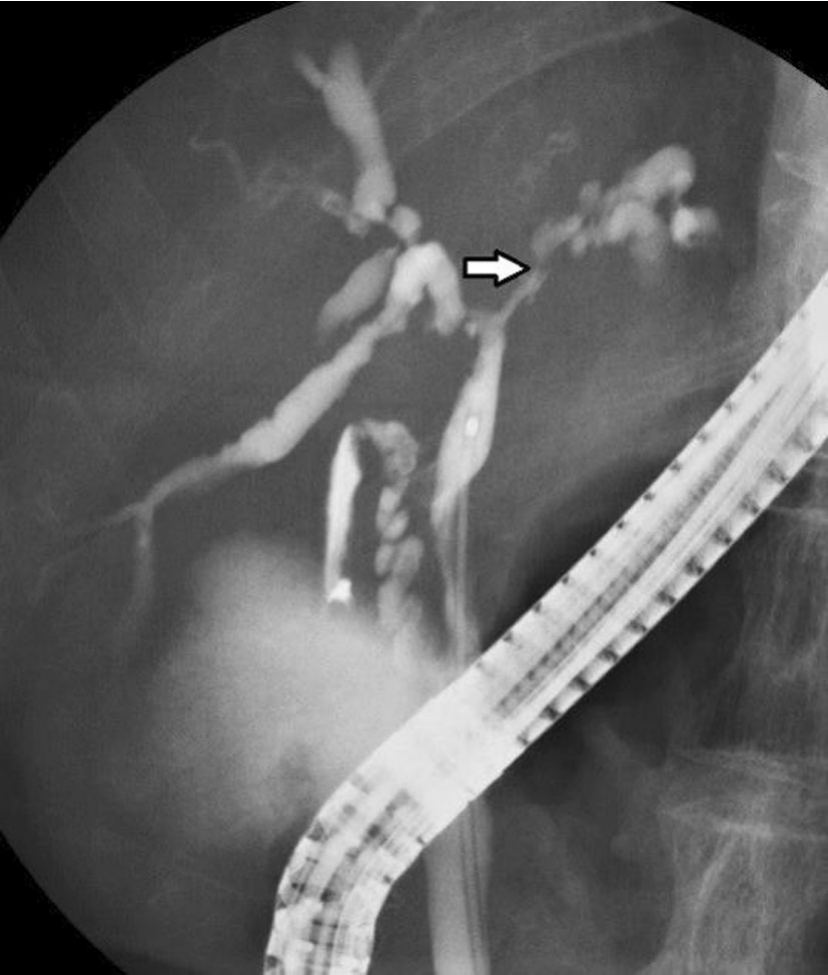
ERCP image of dominant stricture. A bile duct stricture within 2 cm of the branch between the right and left hepatic ducts can be seen

**Fig. 14:**
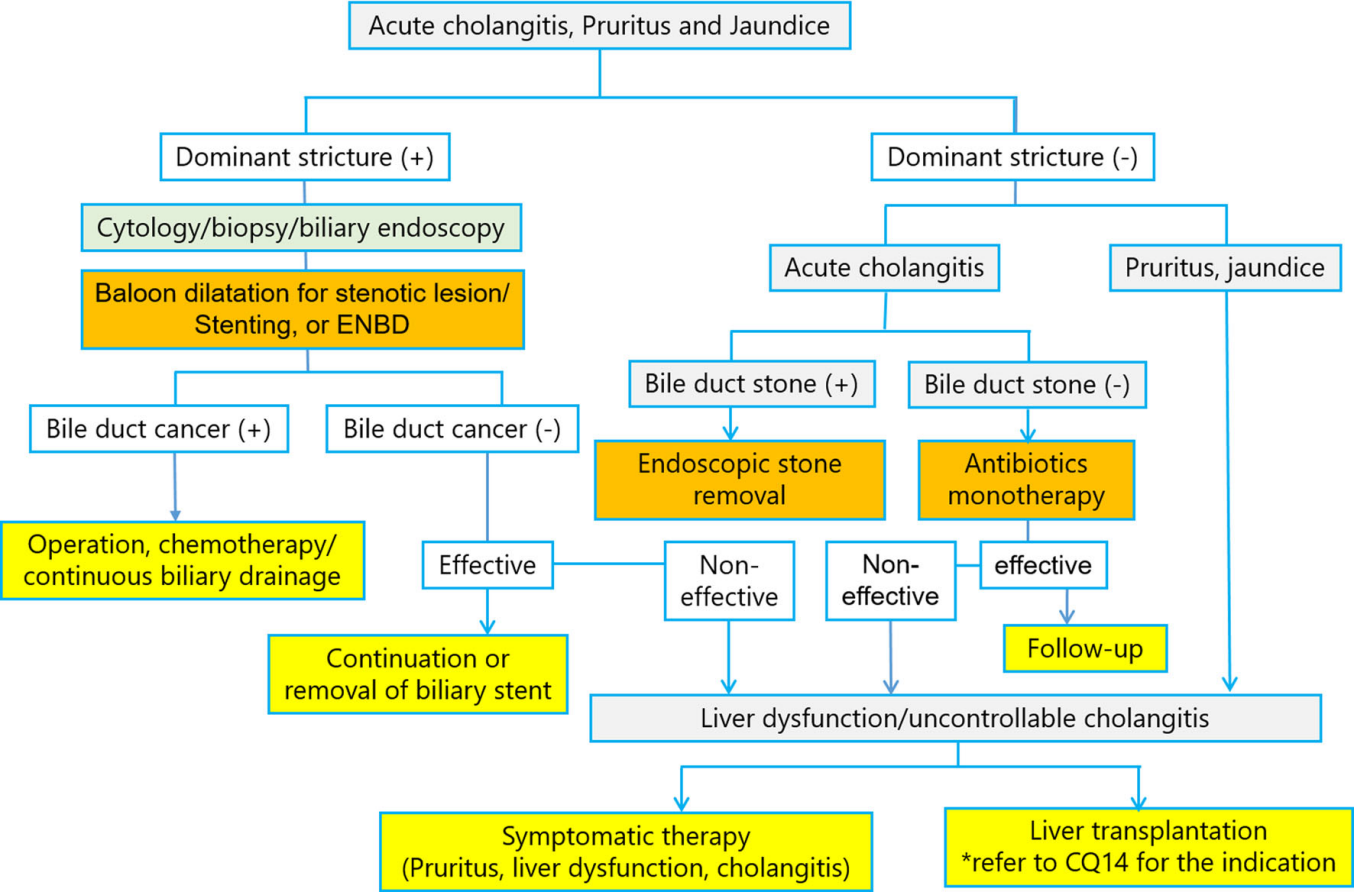
Flowchart for the management of symptomatic PSC

It is difficult to differentiate cholangiocarcinoma that is a complication of PSC from benign bile duct stricture. Contrast enhanced CT cannot be used to differentiate between inflammatory lesions in the bile duct wall and cholangiocarcinoma [114]. Since PET-CT leads to accumulation not only in the cholangiocarcinoma tumor but also results in false positives for inflammatory lesions, the additional use of ERCP and other modalities is required [115–117]. MRCP is non-invasive and useful in the diagnosis of PSC [118]; however, it has difficulties in the differentiation of cholangiocarcinoma using MRCP [119]. Although there are no specific tumor markers, prospective research has indicated that at a CA19-9 cutoff value of 100, sensitivity, specificity, and accuracy are 14, 95, and 61%, respectively [120]. A retrospective analysis reported that when the cutoff value is set at 20 U/ml, sensitivity and specificity are 78 and 67%, respectively. When combined with the use of abdominal sonography, the values are 91 and 62%, respectively, and when combined with CT the values are 100 and 38%, respectively, indicating that although sensitivity increases, specificity declines [121]. Expert opinion recommends that CA19-9 and abdominal sonography/CT were used in combination every 6–12 months as a screening examination for cholangiocarcinoma [48, 121]. ERCP is the recommended examination for dominant strictures (defined as common bile duct strictures 1.5 mm or less in diameter and hepatic duct strictures 1.0 mm or less in diameter that are located within 2 cm of the branch between the right and left hepatic ducts (Fig. 13) [101] that are present at the time of diagnosis or appear during the course of the disease [48]. Bile duct brush cytology for cholangiocarcinoma as a complication of PSC has a sensitivity of 45% (95% CI 35–52%) and a specificity of 97% (95% CI 95–98%). Thus, this low sensitivity is problematic [122]. While sensitivity improves when fluorescence in situ hybridization (FISH) is used in conjunction with brush cytology for the purpose of cytodiagnosis, meta-analysis has shown that the sensitivity is still no more than 68% (95% CI 61–74%) and specificity is no more than 70% (95% CI 66–73%) [123]. Differentiation between benign and malignant bile duct strictures associated with PSC using peroral cholangioscopy (including cholangioscopic biopsy) has been reported to be superior to ERCP [124, 125]. However, as problems exist such as it being impossible to reach the lesion site in some cases or dysplasia diagnosis not being possible [126], this examination is not yet suitable for clinical assessments. Endoscopic evaluation of the dominant stricture was important and was a first step of endoscopic treatment (Fig. 14).

### CQ 11. What pharmacotherapies are effective?


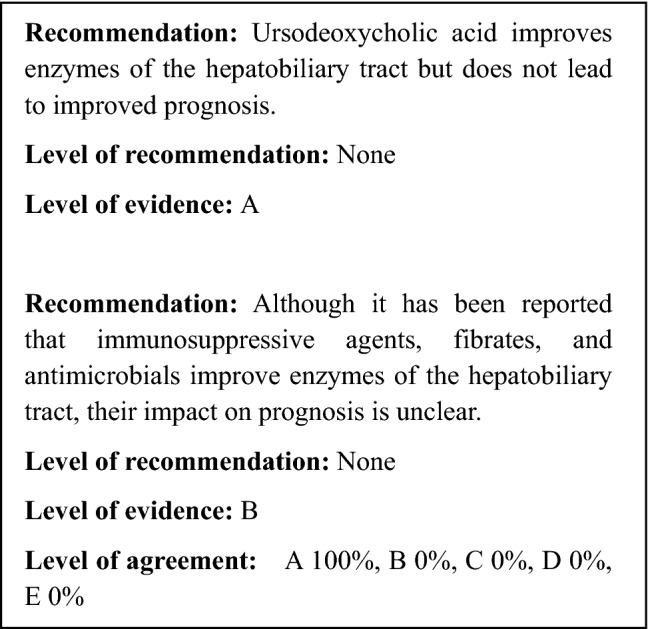


#### Explanation

According to a national survey conducted in Japan, the most commonly used pharmacotherapy is ursodeoxycholic acid, at 76%, followed by steroids (20%) and bezafibrate (20%) [35]. Although bezafibrate is only rarely used overseas, the off-label use of bezafibrate is administered in Japan to cases that are unresponsive to ursodeoxycholic acid. At present, there are no pharmacotherapies that are effective in avoiding death or liver transplantation or in improving prognosis. It has been reported that decreases in ALP levels 1 year after the start of ursodeoxycholic acid administration are linked to long-term prognosis, which is anticipated to indicate that improvement in hepatobiliary tract enzymes leads to improved prognosis [127–130].

1. *Ursodeoxycholic acid* Ursodeoxycholic acid is the pharmacotherapy for PSC that has been researched the most, and numerous random controlled studies (RCT) have reported declines in hepatobiliary tract enzymes [131–140]. It has been reported that high doses (> 15–20 mg/kg) are better at suppressing progression in bile duct profile and hepatic tissue profile findings [136] and tend to improve the Mayo risk score better than low doses [139]. However, the drug is not effective in avoiding endpoints such as death and liver transplantation (risk ratio: 0.66–1.01) [134, 138], and indeed has been reported to increase such risks (risk ratio: 2.3) [140]. Thus, it is currently thought that high doses of ursodeoxycholic acid are not effective. Ursodeoxycholic acid has been subjected to three meta-analysis studies. Although all these showed improvements in hepatobiliary tract enzymes, there was no improvement in prognosis (risk ratio: 0.6–1.04) [141–143]. Three RCT follow-up studies investigated the ability of the drug to prevent colorectal neoplasms (adenoma and cancer). However, the results were contradictory, with some showing that risk declined (risk ratio: 0.26) [144], some showing that risk increased (risk ratio: 4.44) [145], and some showing that risk underwent no change (risk ratio: 0.81) [146]. Two other meta-analysis studies reported no change in the risk of colorectal neoplasms (risk ratio: 0.50–0.87) [147, 148].
2. *Steroids* Steroid therapy has been shown to improve hepatobiliary tract enzymes and the hepatic tissue profile in an uncontrolled prospective study using budesonide [149]. Furthermore, it was shown to have no improvement on hepatobiliary tract enzymes in an RCT using budesonide, while prednisolone alone was shown to improve hepatobiliary tract enzymes [150]. The one meta-analysis study that has been reported concluded that there was no evidence to either recommend or avoid steroid therapy [151]. The results of a case–control study indicated that the efficacy rate was low, at 3.7% [152] and that there were problems with osteoporosis as an adverse effect [149].
3. *Immunosuppressants and immune modulators* An uncontrolled prospective study [153] showed that the immunosuppressant methotrexate improved hepatobiliary tract enzymes, the hepatic tissue profile, and the bile duct profile. However, another uncontrolled prospective study [154] indicated that neither hepatobiliary tract enzymes nor symptoms showed any improvement. An RCT reported that, although there was improvement in hepatobiliary tract enzymes, there was no improvement in the hepatic tissue profile or the bile duct profile [155].
  A case series that studied mycophenolate mofetil reported improvement in hepatobiliary tract enzymes [156], whereas an RCT reported that there was no effect [157].
  Tacrolimus was the object of two uncontrolled prospective studies that reported improvement in hepatobiliary tract enzymes, but continuation of this effect was made difficult by adverse effects [158, 159]. The immune modulators d-penicillamine [160] and colchicine [161, 162] as well as the anti-TNFα antibody infliximab [163] were reported to be ineffective.
4. *Bezafibrate* Based on its mechanism of action, it is anticipated that bezafibrate will be useful against cholestatic diseases [164]. Its effectiveness has been reported by research conducted in Japan, the first of which was a case report published in 2002 [165]. Subsequently, two case series reported improvement in hepatobiliary tract enzymes [166, 167]. In addition, a non-controlled prospective study reported that it was effective in improving hepatobiliary tract enzymes in 64% of the cases studied [168].
5. *Antibiotics* The antibiotics metronidazole [169, 170] and vancomycin [170] were shown to improve hepatobiliary tract enzymes and the Mayo risk score in RCTs. An uncontrolled prospective study of minocycline reported that the drug improved hepatobiliary tract enzymes and the Mayo risk score [171].

### CQ 12. How is pruritus treated?


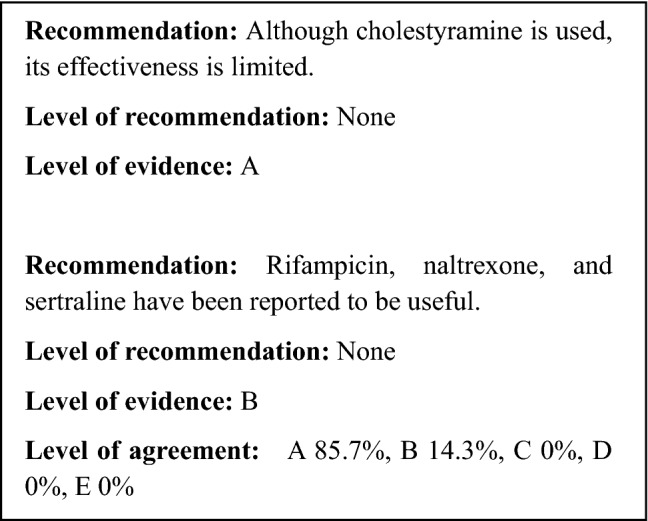


#### Explanation

A national survey conducted in Japan claimed that 17% of PSC patients experience pruritus [35]. There is an extremely small amount of evidence regarding therapies that are effective on the pruritus caused by PSC, although there are a large number of studies on cholestatic diseases as a whole, and especially primary biliary cholangitis (PBC).

The EASL guidelines for cholestatic diseases recommend (1) cholestyramine, (2) rifampicin, (3) naltrexone, and (4) sertraline as therapies for pruritus [55]. However, none of these drugs are covered by the health insurance system in Japan for the indication of pruritus.

In 2015, the kappa opioid receptor agonist nalfurafine was approved for coverage by the health insurance system for use against pruritus experienced by chronic liver disease patients, including those with PSC. However, there is no evidence at this time that it is effective for pruritus associated with PSC.

1. *Anion exchange resins* The anion exchange resin known as cholestyramine has long been reported to reduce pruritus [172], and according to the EASL guidelines, it is the first-line therapy for pruritus [55]. However, meta-analysis has shown that cholestyramine is not effective [173]. An RCT using colesevelam, which has better bile acid absorption than cholestyramine, indicated that it was not effective against pruritus [174]. Thus, there is doubt regarding whether anion exchange resins are effective against pruritus.
2. *Rifampicin* Rifampicin is a type of antibiotic that is also known as rifampin. It works as an agonist on the pregnane X receptor (PXR), which is an orphan nuclear receptor, and it is known to have an induction effect on enzymes that are involved in drug metabolism. The results of a meta-analysis study indicated that it improved pruritus while causing few adverse effects [173].
3. *Opioid antagonists* The results of a meta-analysis study indicated that opioid μ-receptor antagonists (nalmefene, naloxone, naltrexone) are effective against pruritus [173]. Naltrexone is listed as the third-line therapy in the EASL guidelines [55]. However, multiple adverse effects such as withdrawal symptoms such as dizziness, nausea, and headache have been indicated as problems [173].
4. *Selective serotonin reuptake inhibitors* The selective serotonin reuptake inhibitor (SSRI) known as Sertraline was shown in an RCT [175] to be effective. However, another self-controlled study [176] reported that the selective serotonin receptor antagonist known as ondansetron was also effective. Further study of the involvement of serotonin in pruritus therapies is required going forward.
5. *Others* Ursodeoxycholic acid, the most commonly used PSC therapy, was shown to be effective in alleviating pruritus in uncontrolled prospective studies [177, 178], but meta-analysis showed negative results [142]. The steroid budesonide was ineffective, whereas prednisolone was shown to be effective in improving pruritus in an RCT [150]. However, evidence for this is still insufficient. The TNF-α inhibitor etanercept [179] and the antibiotic metronidazole [170] have been reported to be effective, but the evidence is still insufficient for both.

### CQ 13. What are the indications for and method of utilizing biliary drainage?


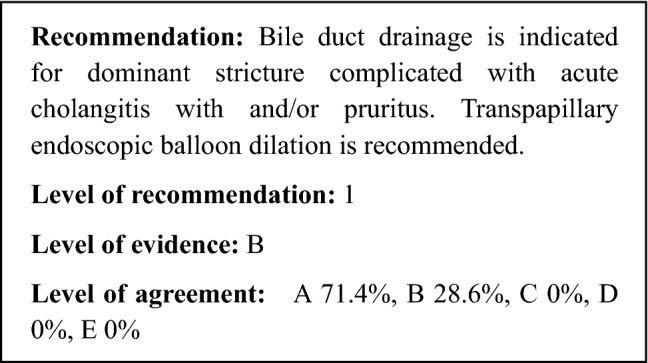


#### Explanation

The dominant strictures that are indicated for drainage are strictures of the common bile duct that are 1.5 mm in diameter or smaller and hepatic duct strictures that are 1.0 mm in diameter or smaller and located within 2 cm of the branch between the right and left hepatic ducts [122]. No prospective comparative studies of endoscopic therapy for dominant stricture have been conducted. Prospective analysis [180–182] has indicated that endoscopic balloon-assisted dilation of the bile duct contributes to improvements in cholangitis, pruritus, hepatic disease, and long-term prognosis. However, endoscopic stricture dilation has also been viewed negatively [122], indicating that the procedure is not effective on all strictures. Navaneethan et al. reported that 34 cases of biliary drainage failure among 294 PSC cases that underwent either stricture dilation or stent placement had cirrhosis of the liver [100]. ERCP has been reported to be effective in the diagnosis and treatment of choledocholithiasis in symptomatic cases [181]. ERCP may be indicated in cases in which symptoms become exacerbated. As it is difficult to differentiate dominant stricture from cholangiocarcinoma with diagnostic imaging alone, cytodiagnosis and other procedures are performed during drainage procedures [183, 184]. Short-term stent placement after stricture dilation is useful and commonly practiced [100]. However, long-term placement often causes complications such as cholangitis and has not confirmed advantages compared to balloon dilation alone [185, 186]. In PSC cases, the high risk of post-ERCP cholangitis makes prophylactic antibiotic administration necessary [73]. Drainage is generally performed using less invasive transpapillary endoscopic therapy [185], but in cases in which this would be difficult to perform, percutaneous drainage and surgical drainage are considered [187, 188]. Flow chart of management of symptomatic PSC is shown in Fig. 14.

### CQ 14. What are the indications for liver transplantation?


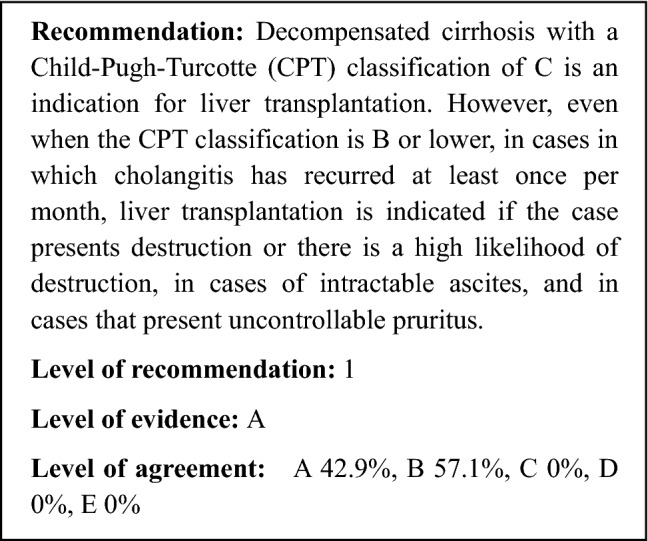


#### Explanation

Liver transplantation is the only definitive treatment for PSC [189]. A recent population-based study conducted in the Netherlands investigated 590 PSC cases and found that the non-liver transplantation rates 10 and 20 years after PSC diagnosis were 78 and 60%, respectively. It was also demonstrated that more severe PSC patients are followed up on by transplantation facilities [20]. This suggests that a large number of PSC patients require liver transplantation and that it is important to refer PSC patients to transplantation facilities. A national survey of 428 PSC patients in Japan reported that 54 cases (13%) underwent liver transplantation [57].

The Model for End-Stage Liver Disease (MELD) score, which is also used for decompensated cirrhosis caused by other diseases, and the Child–Pugh–Turcotte (CPT) classification are used as the indication criteria for liver transplantation in cases of PSC. In addition to converting liver function into a comprehensive score, other indications unique to PSC include recurrent cholangitis and uncontrollable pruritus. In cases in which these cause patient quality of life (QOL) to decrease markedly, liver transplantation is indicated even if hepatic reserved is maintained [54, 55, 190]. In particular, it is necessary to comprehensively determine the timing of liver transplantation in cases of liver transplantation from a living donor when there is a one-to-one correspondence between the donor and the recipient. IBD comorbidity will not have a negative effect on the liver transplantation, but it is desirable to use pharmacotherapy to ensure remission or to perform transplantation on cases that have already completed surgical treatment for IBD.

Brain-dead liver transplantation is indicated in cases of decompensated cirrhosis with a CPT score of 10 or above. After being registered on the brain-dead liver transplantation waiting list, brain-dead donor livers are assigned in order of the highest MELD scores [191]. In light of the fact that there are many PSC cases that experience recurrent cholangitis in spite of maintaining hepatic reserve, the following indications are to be added:

1. Selection is made according to the selection criteria for decompensated cirrhosis and the order of registration.
2. However, in cases in which the patient experiences recurrent cholangitis once per month or more frequently, the patient is registered with the equivalent of an MELD score of 16. Then, brain-dead donor liver transplantation is carried out in order of the MELD score as the disease progresses. The equivalent score is assigned at 16 even if the actual MELD score is lower than 16. Since cholangioma complication is a problem when there are intrahepatic nodules, the physician is required to clearly indicate accurate liver dynamic CT and MRI findings.
3. In pediatric cases (under the age of 18 at onset), an application is submitted once the liver cirrhosis CPT score reaches 7 or above (equivalent to 10) and an MELD score equivalent of 16 is assigned at the time of registration. Two points are added every 6 months after registration.

In Japan, over 90% of liver transplantations are live donor transplantations, and the timing and indications for liver transplantation are more flexible than elsewhere. In cases of liver donor liver transplantation when there is a one-to-one correspondence between the donor and recipient, there is no need to consider ensuring fairness to patients with decompensated cirrhosis caused by other diseases. Cases that present pre-symptoms of decompensation, such as rupture of esophageal varices and intractable ascites, and cases with prominent symptoms, such as recurrent cholangitis and uncontrollable pruritus, are indicated for liver transplantation even if their CPT classification is B or the equivalent. A national survey of live donor transplantation for PSC in Japan demonstrated that the PSC recurrence rate was high and the therapeutic outcome was poor in cases with high MELD scores (≥ 24) and first-degree relative donors [192].

The issue of recurrence of the underlying disease (PSC) is important in the issue of liver transplantation for PSC [192]. Both brain-dead liver transplantation and live donor liver transplantation have a high recurrence rate, but the frequency is higher in the latter case, at approximately one in three, and eventual re-transplantation is also required in many cases [193, 194]. However, improvements in QOL can be expected over the long-term if appropriate management is practiced even in cases of recurrence. Thus, although liver transplantation is a significant treatment, it is necessary to obtain informed consent from both the patient and the donor prior to carrying out the transplantation.

### CQ 15. What is the prognosis for primary sclerosing cholangitis?


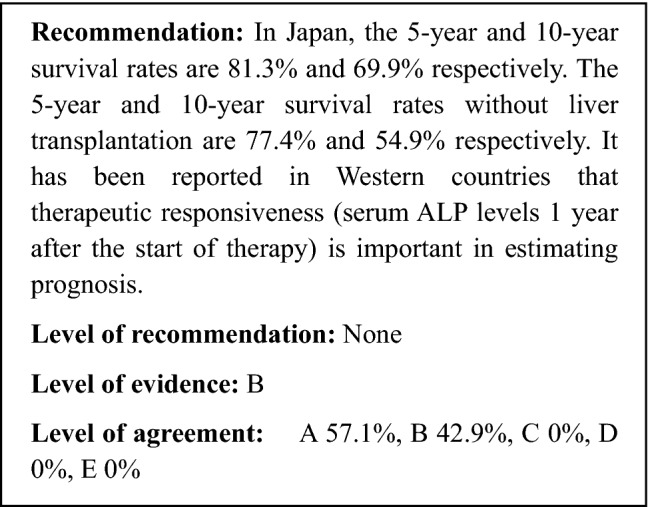


#### Explanation

**Fig. 15:**
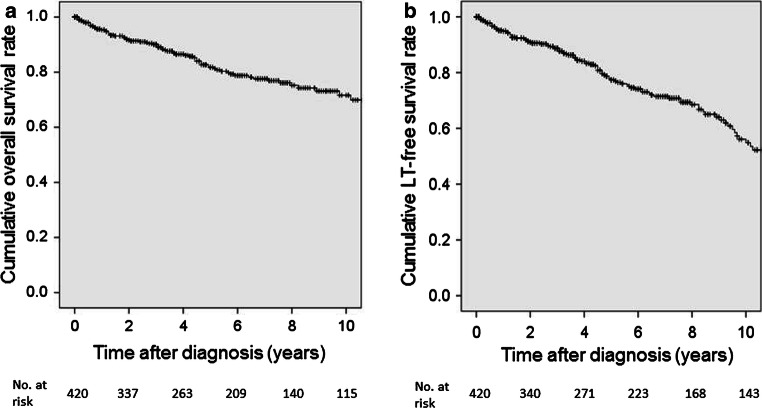
Prognosis of PSC patients in Japan (Modified citation from Ref. [30]). **a** Overall survival. **b** Overall survival without liver transplantation

The natural history of PSC is complex with a variety of patterns ranging from those who experience little disease advancement in a year or more to those who experience rapid decline. In general, however, the disease advances gradually, with blood test results worsening and then improving and clinical symptoms repeatedly disappearing and reappearing as the disease eventually develops into cirrhosis of the liver. A national survey conducted in Japan reported that among 435 registered cases, the 5- and 10-year survival rates were 81.3 and 69.9%, respectively, and the 5- and 10-year survival rates in cases that did not undergo liver transplantation were 77.4 and 54.9%, respectively (Fig. 15) [30].

**Fig. 16:**
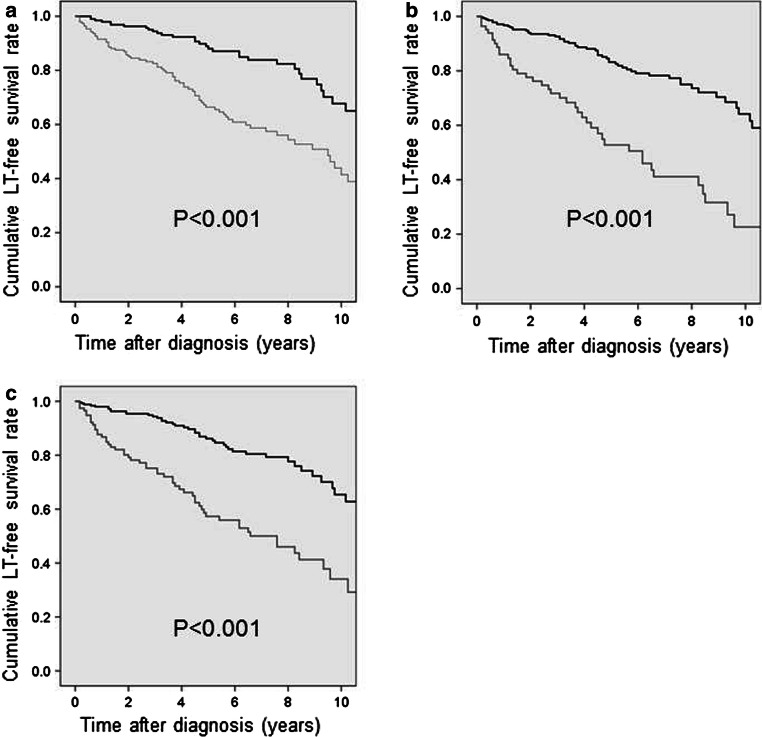
Prognosis of PSC patients in Japan: Stratification based on prognostic factors. (Modified citation from a 2015 National Survey on PSC: Ref. [30]). **a** Age at diagnosis: bold line, < 44 years; thin line, ≥ 44 years. **b** Serum albumin level at diagnosis: bold line, ≥ 3.5 g/dl; thin line, < 3.5 g/dl. **c** Total bilirubin level at diagnosis: bold line, < 1.5 mg/dl; thin line, ≥ 1.5 mg/dl

A variety of prognosis predictive factors were reported in the past. The main factors are age at the time of diagnosis [53, 56, 111, 195–198], bilirubin [53, 56, 111, 196, 198, 199], albumin [56, 200], CPT score [197], hepatic fibrosis [53, 111, 195], and splenohepatomegalia [56, 195], among others. The results of a national survey conducted in Japan in 2003 indicated that the two factors of age at diagnosis (young) and low bilirubin [198] were related to good prognosis, and a single-facility analysis reported that low serum ALP levels during disease progression was related to good prognosis [201]. Repeated multivariate analysis performed on the results of a national survey conducted in 2015 also showed that age at diagnosis (young), low bilirubin levels, and high albumin levels were related to good prognosis (Fig. 16) [30]. Therefore, cases that do not present jaundice and young cases have relatively good prognosis. And when restricted to cases that were asymptomatic at diagnosis, the 5- and 10-year survival rates were shown to be 87.3 and 66.5%, respectively. These improved to 91.3 and 73.5%, respectively, in cases that were both asymptomatic at diagnosis and young (younger than 44 years). Although only a small number of cases have been studied in Japan, a single-facility study indicated that elderly patients actually have a good prognosis [38].

Studies of the prognostic factors for PSC that indicated that PSC cases with high serum IgG4 levels progress poorly with an unfavorable prognosis have mainly been conducted in the United States [202, 203]. A subsequent study conducted in Europe had a negative view of the influence of IgG4 [204]. Analysis of the results of a national survey conducted in Japan in 2015 indicated that there was no connection between IgG4 levels and the pathophysiology and prognosis of PSC [30]. As studies that indicate a connection between IgG4 levels and PSC prognosis, etc. include very old case data, IgG4-SC cases may have been included.

In recent years, the development of novel drugs for the treatment of PSC and clinical trials has been planned. However, since arrival at hard endpoints such as death and liver transplantation requires long periods of time, the fact that trials generally use alternative endpoints has been pointed out as a procedural problem. Retrospective studies have reported that there is a relation between ALP levels 1 year after the start of therapy and long-term prognosis [127–130]. However, since there are patients who present increases and decreases in ALP levels during the natural course of PSC, serum ALP levels alone are problematic when used as an alternative endpoint. Examination of discussions among PSC researchers in Western countries indicates statements suggesting that the combination of serum ALP levels and the non-invasive assessment of fibrosis could be used as a valid alternative endpoint [205].

### CQ 16. What are the complications associated with primary sclerosing cholangitis?


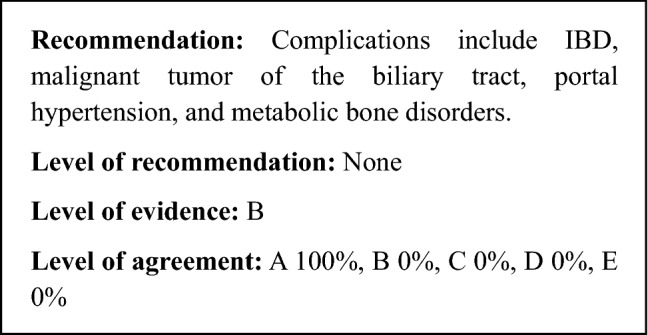


#### Explanation

In Western countries, 60–80% of PSC cases are complicated with inflammatory bowel diseases (IBD) [48, 54]. A national survey conducted in Japan placed that figure at 40%, but when restricted to younger patients, the figure was 61%, which is on par with Western countries [30]. IBD is often diagnosed after PSC diagnosis and the lower endoscopy is recommended regardless of whether the patient presents symptoms of IBD [48, 54]. Ulcerative colitis (UC) as a complication of PSC is characterized by the lack of rectal lesions and the frequency with which backwash ileitis is observed [81]. The risk of onset of colorectal neoplasia (dysplasia, carcinoma) associated with UC is significantly higher in cases of UC in association with PSC than with UC alone [206]. However, the frequency of onset in Japan is currently unclear.

The annual rate of cholangiocarcinoma is 1–2% and the lifetime rate is 5–14% [207, 208]. It commonly occurs within 1–3 years after diagnosis [117]. Several retrospective analyses of cholecystectomy indicated that gallbladder cancer was present in 7–21% of the cases, and the rate of complication with dysplasia was high [66, 208–210]. Thus, cholecystectomy is recommended in cases of torose lesions of the gallbladder that are 8–10 mm in size or larger despite this procedure being known to be frequently associated with postoperative complications [208, 209].

Acute cholangitis with bile duct stricture has been reported in 6.1% of cases at the time of PSC diagnosis, and it is known that recurrent cholangitis due to dominant stricture causes reduced hepatic function and leads to the need for earlier liver transplantation [24, 48]. Advancing cirrhosis of the liver causes portal hypertension as well as esophageal and gastric venous varices. Thus, it is recommended that upper endoscopic screening was performed when the platelet count is at 140,000–200,000 or below [211–213]. Metabolic bone diseases (osteoporosis, osteopenia) due to cholestatic cirrhosis of the liver occur in 4–10% of cases, suggesting that advancing age and cirrhosis of the liver are related factors [48, 54, 214].

## Conclusion

We had made 16 guidelines about Epidemiology/Pathophysiology, Diagnostics, Therapy and Prognosis. Also, we had made both diagnostic and therapeutic flow chart. We hope that these guidelines will contribute to the improvement and development of the medical care of PSC.
