# Correction to: Clinical guidelines for primary sclerosing cholangitis 2017

**DOI:** 10.1007/s00535-022-01867-7

**Published:** 2022-03-16

**Authors:** Hiroyuki Isayama, Susumu Tazuma, Norihiro Kokudo, Atsushi Tanaka, Toshio Tsuyuguchi, Takahiro Nakazawa, Kenji Notohara, Suguru Mizuno, Nobuhisa Akamatsu, Masahiro Serikawa, Itaru Naitoh, Yoshiki Hirooka, Toshifumi Wakai, Takao Itoi, Tomoki Ebata, Shinji Okaniwa, Terumi Kamisawa, Hiroki Kawashima, Atsushi Kanno, Keiichi Kubota, Masami Tabata, Michiaki Unno, Hajime Takikawa, PSC guideline committee Members: Ministry of Health, Labour and Welfare (Japan) Research Project, The Intractable Hepatobiliary Disease Study Group

**Affiliations:** 1 Department of Gastroenterology, Graduate School of Medicine, Juntendo University, Tokyo, Japan; 2 Department of General Internal Medicine, Graduate School of Biomedical and Health Sciences, Hiroshima University Hospital, 1-2-3, Kasumi, Minami-ku, Hiroshima, 734-8551 Japan; 3 Department of Surgery, National Center for Global Health and Medicine, Tokyo, Japan; 4 Department of Medicine, Teikyo University School of Medicine, Tokyo, Japan; 5 Department of Medicine and Gastroenterology, Chiba University, Chiba, Japan; 6 Department of Gastroenterology, Japanese Red Cross Nagoya Daini Hospital, Nagoya, Japan; 7 Department of Anatomic Pathology, Kurashiki Central Hospital, Kurashiki, Japan; 8 Department of Gastroenterology, Graduate School of Medicine, The University of Tokyo, Tokyo, Japan; 9 Artificial Organ and Transplantation Division, Department of Surgery, Graduate School of Medicine, The University of Tokyo, Tokyo, Japan; 10 Department of Gastroenterology and Metabolism, Applied Life Sciences, Institute of Biomedical and Health Sciences, Hiroshima University, Hiroshima, Japan; 11 Department of Gastroenterology and Metabolism, Graduate School of Medical Sciences, Nagoya City University, Nagoya, Japan; 12 Department of Endoscopy, Nagoya University Hospital, Nagoya, Japan; 13 Division of Digestive and General Surgery, Niigata University Graduate School of Medical and Dental Sciences, Niigata, Japan; 14 Department of Gastroenterology and Hepatology, Tokyo Medical University, Tokyo, Japan; 15 Division of Surgical Oncology, Department of Surgery, Nagoya University Graduate School of Medicine, Nagoya, Japan; 16 Department of Gastroenterology, Iida Municipal Hospital, Nagano, Japan; 17 Department of Internal Medicine, Tokyo Komagome Metropolitan Hospital, Tokyo, Japan; 18 Department of Gastroenterology, Nagoya University Graduate School of Medicine, Nagoya, Japan; 19 Division of Gastroenterology, Tohoku University Graduate School of Medicine, Sendai, Miyagi Japan; 20 Second Department of Surgery, Dokkyo Medical University, Tochigi, Japan; 21 Department of Surgery, Matsusaka Central General Hospital, Matsusaka, Mie Japan; 22 Department of Surgery, Tohoku University Graduate School of Medicine, Sendai, Miyagi Japan

## Correction to: J Gastroenterol (2018) 53:1006–1034 10.1007/s00535-018-1484-9

The article “Clinical guidelines for primary sclerosing cholangitis 2017”, written by Hiroyuki Isayama, Susumu Tazuma, Norihiro Kokudo, Atsushi Tanaka, Toshio Tsuyuguchi, Takahiro Nakazawa, Kenji Notohara, Suguru Mizuno, Nobuhisa Akamatsu, Masahiro Serikawa, Itaru Naitoh, Yoshiki Hirooka, Toshifumi Wakai, Takao Itoi, Tomoki Ebata, Shinji Okaniwa, Terumi Kamisawa, Hiroki Kawashima, Atsushi Kanno, Keiichi Kubota, Masami Tabata, Michiaki Unno, Hajime Takikawa, PSC guideline committee Members: Ministry of Health, Labour and Welfare (Japan) Research Project, The Intractable Hepatobiliary Disease Study Group, was originally published Online First without Open Access. After publication in volume 53, issue 9, page 1006–1034 the author decided to opt for Open Choice and to make the article an Open Access publication. Therefore, the copyright of the article has been changed to © The Authors 2021 and the article is forthwith distributed under the terms of the Creative Commons Attribution 4.0 International License, which permits use, sharing, adaptation, distribution and reproduction in any medium or format, as long as you give appropriate credit to the original author(s) and the source, provide a link to the Creative Commons licence, and indicate if changes were made. The images or other third party material in this article are included in the article's Creative Commons licence, unless indicated otherwise in a credit line to the material. If material is not included in the article's Creative Commons licence and your intended use is not permitted by statutory regulation or exceeds the permitted use, you will need to obtain permission directly from the copyright holder. To view a copy of this licence, visit http://creativecommons.org/licenses/by/4.0/. The original article has been corrected.

